# Immunotherapy in urological cancers: new paradigms and a systematic review of clinical trials and real-world evidence

**DOI:** 10.3389/fonc.2026.1871832

**Published:** 2026-08-12

**Authors:** Nabil Ismaili, Sanaa El Majjaoui

**Affiliations:** 1 Department of Medical Oncology, Mohammed VI Faculty of Medicine, Mohammed VI University of Sciences and Health (UM6SS), Mohammed VI Foundation of Sciences and Health (FM6SS), Casablanca, Morocco; 2 Oncopathology, Biology and Environment of Cancer Laboratory, Mohammed VI Center for Research and Innovation (CM6RI), Mohammed VI Foundation of Sciences and Health (FM6SS), Rabat, Morocco; 3 Department of Radiotherapy, Mohammed VI Faculty of Medicine, Mohammed VI University of Sciences and Health (UM6SS), Mohammed VI Foundation of Sciences and Health (FM6SS), Casablanca, Morocco

**Keywords:** antibody-drug conjugates, bladder cancer, cancer vaccines, immune checkpoint inhibitors, immunotherapy, kidney cancer, prostate cancer, radiotherapy

## Abstract

**Introduction:**

The therapeutic landscape of urological cancers has undergone a paradigm shift with the advent of immunotherapy. This systematic review synthesizes clinical trials that have established new standards of care, complemented by evidence on immunotherapy-radiotherapy combinations and real-world data.

**Methods:**

A comprehensive search of PubMed/MEDLINE and Embase (2010–2026) identified phase II/III trials evaluating ICIs, ADCs, vaccines, or cellular therapies, as well as immunoradiotehrapy, observational studies, and registries reporting real-world outcomes.

**Results:**

Fifty-three clinical trials met the inclusion criteria: bladder cancer (n=14), kidney cancer (n=21), and prostate cancer (n=18), complemented by immunoradiotherapy trials (n=20), and real-world evidence (n=19). In bladder cancer, perioperative durvalumab and Enfortumab Vedotin (EV) plus pembrolizumab improved outcomes in MIBC, and the EV+pembrolizumab combination became a frontline standard for metastatic disease. Disitamab vedotin plus toripalimab improved outcomes in HER2-expressing tumours, and ctDNA-guided adjuvant atezolizumab introduces precision therapy for molecular residual disease. Emerging immunoradiotherapy combinations showed promising bladder-sparing potential (CR rates 64-88%). In kidney cancer, dual ICI and ICI+TKI combinations demonstrated long-term survival benefits. The RAMPART trial introduced adjuvant durvalumab ± tremelimumab, while transcriptomic-guided therapy and treatment of rare translocation RCC emerged from 2025–2026 trials. In prostate cancer, sipuleucel-T remains the first approved cancer vaccine, while newer trials explored ICIs (durvalumab+tremelimumab) and personalized peptide vaccines. Biomarker-driven approaches emerged across all tumor types, including ctDNA-guided therapy in bladder cancer, KIM 1 in kidney cancer, and PD-L1/DDR status in prostate cancer. Immunoradiotherapy combinations demonstrated activity in mCRPC (CA184-043, 5-year OS 7.9% vs 2.7%) and oligometastatic RCC (RAPPORT, ORR 63%). Real-world evidence confirmed trial findings while revealing critical gaps in access, the prognostic dominance of performance status, and the potential for radiotherapy-immunotherapy synergy.

**Conclusion:**

Immunotherapy has become a cornerstone of urological oncology. Current paradigms include early ICI/ADC intensification in bladder cancer, ICI-based combinations in renal cell carcinoma, and the gradual integration of vaccines and checkpoint inhibitors in prostate cancer. Real-world evidence and immunoradiotherapy represents an emerging frontier, though optimal fractionation and sequencing require further investigation. Despite major advances, challenges remain in overcoming resistance, optimizing sequencing, and ensuring equitable access.

## Graphical Abstract

**Figure:**
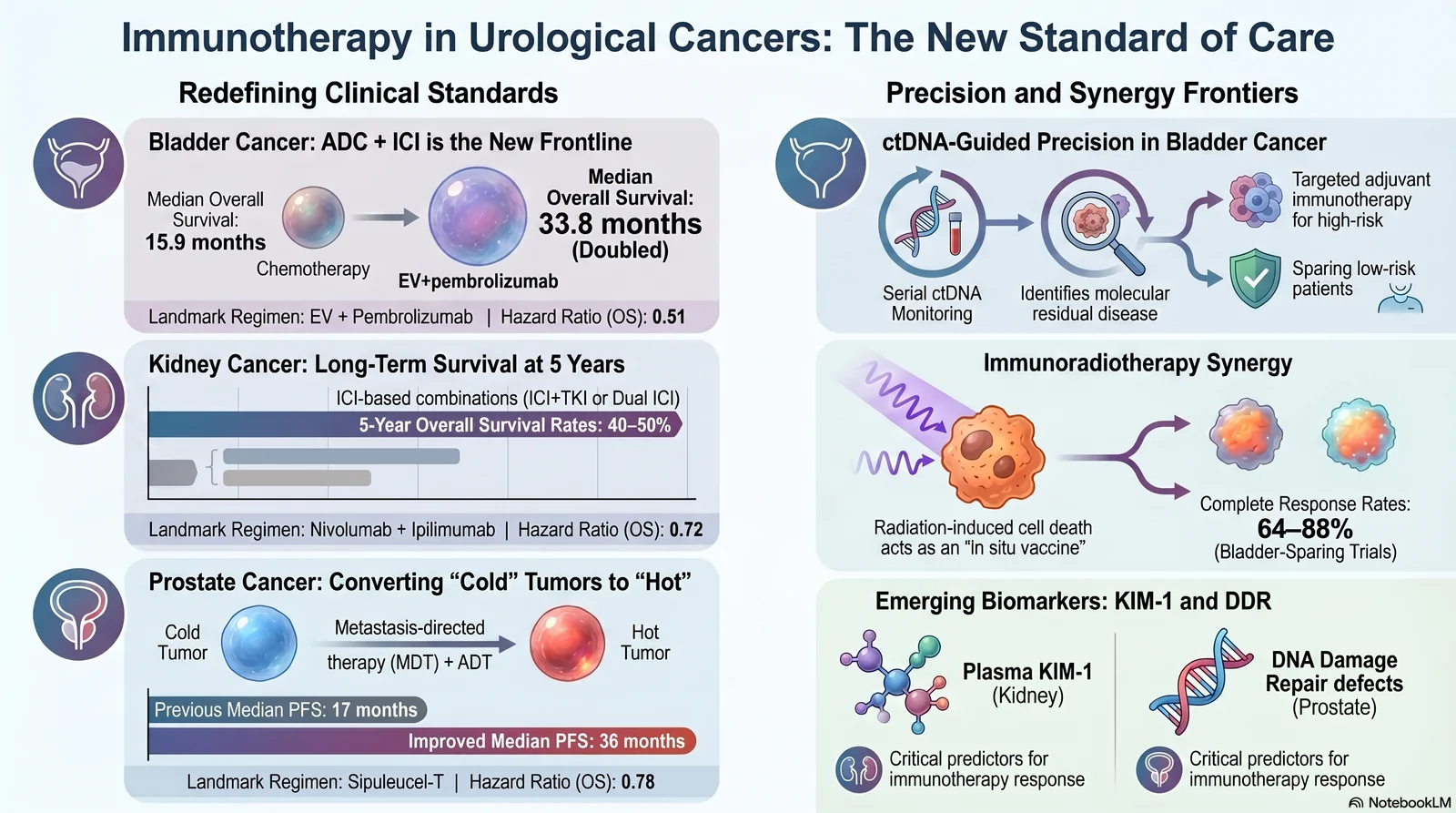
Provide the Systematic Review Registration details (Unique Identifier and URL of the publicly accessible website), if applicable.

## 1 Introduction

The field of genitourinary (GU) oncology has seen transformative developments over the past decade and a half. Historically, treatment options for advanced urological cancers were limited to cytotoxic chemotherapy, cytokine therapy (interleukin-2, interferon-α), and androgen deprivation therapy. However, the therapeutic landscape has been fundamentally reshaped by the advent of immunotherapy, beginning with the first FDA-approved cancer vaccine (sipuleucel-T) in 2010 and accelerating with the approval of immune checkpoint inhibitors (ICIs) targeting PD-1/PD-L1 and CTLA-4 (1, 2).

### 1.1 Biological rationale for immunotherapy in urological cancers

The biological rationale for immunotherapy in urological cancers stems from the unique immunobiology of each tumor type. Bladder cancer, particularly urothelial carcinoma, is characterized by one of the highest somatic mutation frequencies among solid tumors, second only to melanoma and lung cancer. This high tumor mutational burden (TMB) results from exposure to environmental carcinogens and endogenous mutational processes, generating numerous neoantigens that can be recognized by the immune system. The success of intravesical BCG immunotherapy in non-muscle-invasive bladder cancer, first introduced in the 1970s, provided early proof-of-principle that immune activation could control urothelial malignancies. BCG induces a local inflammatory response characterized by Th1 polarization, recruitment of CD4+ and CD8+ T cells, and activation of natural killer (NK) cells, leading to antitumor effects (3).

Renal cell carcinoma (RCC) has long been considered immunologically distinct among solid tumors. Spontaneous regressions of metastatic RCC, though rare, have been documented, suggesting inherent immunogenicity. The classical response to high-dose interleukin-2 (IL-2) therapy in a subset of patients provided the first evidence that immune manipulation could produce durable complete responses in metastatic RCC. The molecular basis for RCC immunogenicity is multifactorial: inactivation of the von Hippel-Lindau (VHL) tumor suppressor gene in clear cell RCC leads to constitutive activation of hypoxia-inducible factors (HIFs), which drive expression of vascular endothelial growth factor (VEGF) and other angiogenic factors. This angiogenic phenotype is accompanied by infiltration of various immune cell populations, including tumor-associated macrophages (TAMs), myeloid-derived suppressor cells (MDSCs), and regulatory T cells (Tregs), creating a complex immunosuppressive microenvironment that can be reversed by immune checkpoint inhibition (4, 5).

Prostate cancer presents a different immunobiological challenge. Historically considered an immunologically “cold” tumor, prostate cancer has a relatively low somatic mutation rate and limited T-cell infiltration. However, recent evidence has revealed that androgen deprivation therapy (ADT) can enhance T-cell infiltration into the tumor microenvironment, potentially converting “cold” tumors into “hot” tumors amenable to checkpoint inhibition. ADT induces a counter-regulatory influx of immunosuppressive Tregs, providing a rationale for combining ADT with Treg-depleting agents such as anti-CTLA-4 antibodies. Furthermore, prostate cancer expresses several tissue-restricted antigens, including prostatic acid phosphatase (PAP) and prostate-specific antigen (PSA), which have been successfully targeted by cancer vaccines (2, 6).

### 1.2 Mechanisms of action of immunotherapeutic agents

#### 1.2.1 Immune checkpoint inhibitors

ICIs are monoclonal antibodies that block inhibitory receptors or ligands on T cells or antigen-presenting cells, thereby unleashing antitumor immune responses. The clinically most relevant checkpoints are cytotoxic T-lymphocyte-associated protein 4 (CTLA-4) and the programmed cell death protein 1 (PD-1) pathway.

CTLA-4 is expressed on activated T cells and regulatory T cells, where it competes with the co-stimulatory receptor CD28 for binding to CD80/CD86 on antigen-presenting cells. By delivering an inhibitory signal, CTLA-4 raises the threshold for T-cell activation, particularly in lymph nodes during the priming phase. Anti-CTLA-4 antibodies (ipilimumab, tremelimumab) block this interaction, promoting T-cell priming and expansion. Importantly, anti-CTLA-4 antibodies can also deplete tumor-infiltrating Tregs through antibody-dependent cellular cytotoxicity (ADCC) when engineered with appropriate Fc regions, as demonstrated with the Fc-enhanced anti-CTLA-4 antibody (7, 8).

PD-1 is expressed on activated T cells, and its ligands PD-L1 and PD-L2 are often upregulated on tumor cells and immune cells within the tumor microenvironment. Engagement of PD-1 by its ligands delivers an inhibitory signal that limits T-cell effector function, promoting T-cell exhaustion. Anti-PD-1 antibodies (nivolumab, pembrolizumab) and anti-PD-L1 antibodies (atezolizumab, avelumab, durvalumab) block this interaction, reinvigorating exhausted T cells and restoring their cytotoxic capacity (7).

The combination of CTLA-4 and PD-1 blockade has shown synergistic effects in preclinical models and clinical trials. CTLA-4 blockade promotes the priming and expansion of tumor-specific T cells, while PD-1 blockade prevents their exhaustion in the tumor microenvironment, resulting in enhanced antitumor activity.

#### 1.2.2 Antibody drug conjugates

ADCs represent an innovative class of targeted immunotherapies that combine the specificity of monoclonal antibodies with the potency of cytotoxic payloads. The antibody component recognizes tumor-associated antigens, delivering the cytotoxic drug directly to cancer cells while minimizing systemic toxicity. Upon binding to its target, the ADC is internalized and trafficked to lysosomes, where the linker is cleaved and the payload is released.

Enfortumab vedotin (EV) targets Nectin-4, a cell adhesion molecule highly expressed in urothelial carcinoma. The antibody is conjugated to monomethyl auristatin E (MMAE), a potent microtubule-disrupting agent, via a protease-cleavable linker. Upon internalization, MMAE is released, inducing cell cycle arrest and apoptosis. Importantly, MMAE can diffuse across cell membranes, killing neighboring tumor cells regardless of their Nectin-4 expression, a phenomenon known as the bystander effect (9, 10).

Disitamab vedotin targets HER2 and incorporates MMAE as its payload. Sacituzumab govitecan targets Trop-2 and delivers SN-38, the active metabolite of irinotecan, via a hydrolyzable linker. These ADCs have shown remarkable activity in urothelial carcinoma, both as monotherapy and in combination with ICIs (2, 11).

#### 1.2.3 Cancer vaccines

Cancer vaccines are designed to stimulate the patient’s own immune system to recognize and attack tumor cells. Unlike preventive vaccines, therapeutic cancer vaccines are administered to patients with established cancer.

Sipuleucel-T is an autologous cellular immunotherapy that remains the only FDA-approved cancer vaccine for prostate cancer. It is manufactured by culturing a patient’s peripheral blood mononuclear cells (PBMCs) with PA2024, a recombinant fusion protein consisting of prostate acid phosphatase (PAP) linked to granulocyte-macrophage colony-stimulating factor (GM-CSF). This process activates antigen-presenting cells (APCs), particularly dendritic cells, which then present PAP-derived peptides to T cells. The activated cellular product is reinfused in three doses at two-week intervals. Sipuleucel-T induces both cellular and humoral immune responses against PAP and, through antigen spread, against secondary tumor antigens including PSA, KLK2, and galectin-3 (12).

Personalized peptide vaccines, such as those tested in Japanese trials, select up to four peptides from a warehouse of cancer-associated antigens based on each patient’s HLA type and pre-existing peptide-specific IgG levels. This approach aims to overcome the heterogeneity of immune responses and maximize vaccine immunogenicity.

Telomerase-based vaccines (GX301) target human telomerase reverse transcriptase (hTERT), a universal tumor-associated antigen expressed in most cancers. GX301 comprises four hTERT peptides and two adjuvants (Montanide ISA-51 VG and imiquimod), administered intradermally (13).

#### 1.2.4 Adoptive cellular therapies

Adoptive cell transfer involves the ex vivo expansion and/or engineering of a patient’s own immune cells before reinfusion. While chimeric antigen receptor (CAR)-T cells have revolutionized the treatment of hematologic malignancies, their application in solid tumors remains challenging. Cytokine-induced killer (CIK) cells, expanded from PBMCs with IL-2, anti-CD3 antibody, and interferon-γ, have been evaluated in urological cancers with modest success (14).

**Figure 1:**
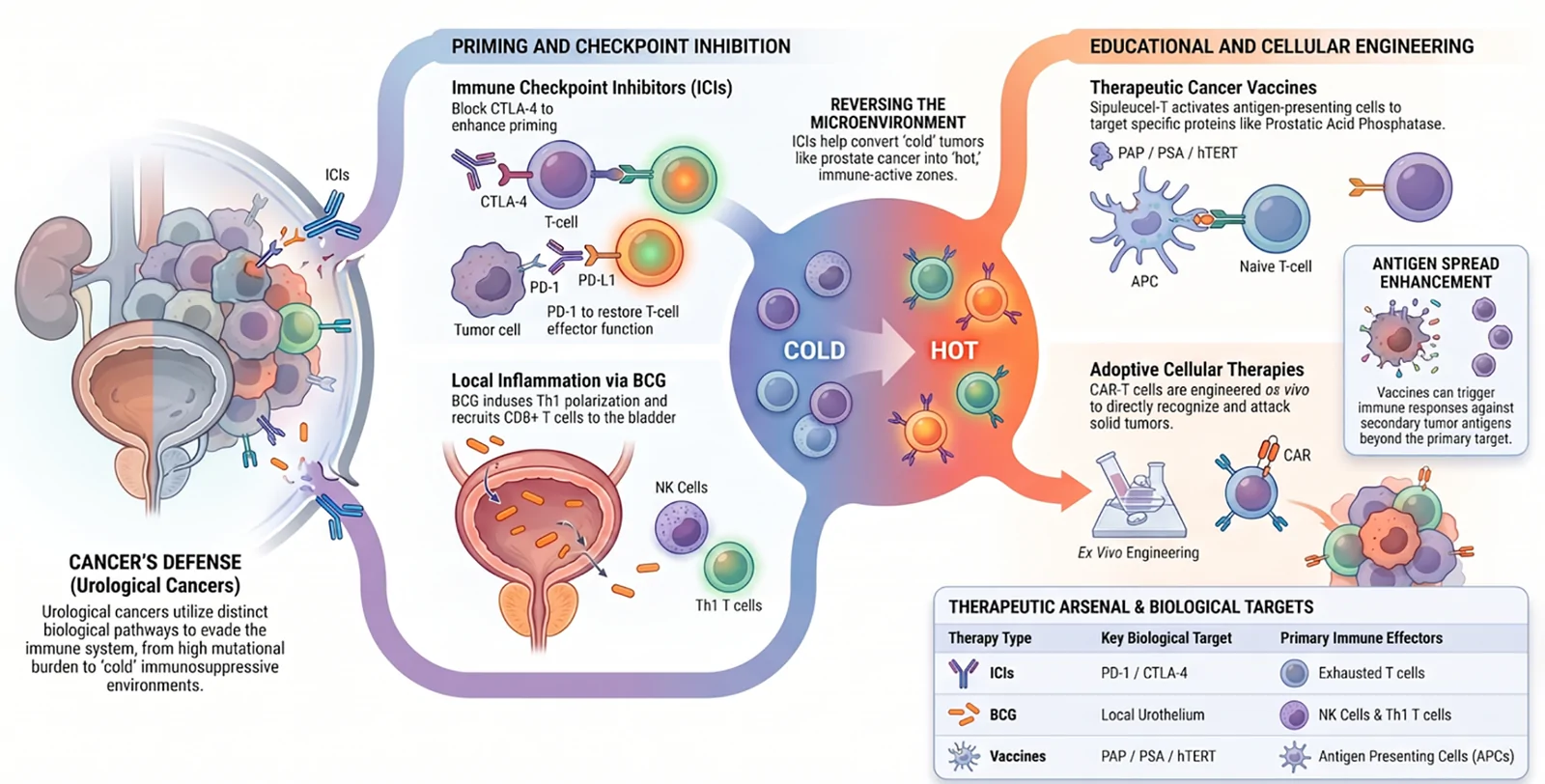
Mechanistic landscape of immunotherapeutic classes and evolving paradigms in urological malignancies. This schematic illustrates the principal immunotherapeutic strategies and their biological underpinnings across bladder, kidney, and prostate cancers. Upper panel (Priming and Checkpoint Inhibition): ICIs block CTLA-4 to enhance T-cell priming in lymph nodes, and PD-1/PD-L1 to restore effector function of exhausted T cells within the tumor microenvironment. Intravesical BCG therapy induces local inflammation characterized by Th1 polarization, CD8+ T-cell recruitment, and NK cell activation. Urological cancers employ distinct immune evasion mechanisms, ranging from high tumor mutational burden (bladder cancer) to immunosuppressive “cold” microenvironments (prostate cancer). Middle panel (Educational and Cellular Engineering): Therapeutic cancer vaccines, such as sipuleucel-T, activate antigen-presenting cells (APCs) to present tumor-associated antigens (PAP, PSA, hTERT), promoting T-cell priming and antigen spread to secondary tumor antigens. Adoptive cellular therapies, including chimeric antigen receptor (CAR)-T cells, are engineered ex vivo to directly recognize and attack solid tumors. Lower panel (Therapeutic Arsenal and Biological Targets): Summary table linking each therapy class to its key biological target and primary immune effector cells. This framework contextualizes the clinical trial evidence presented in subsequent sections. Abbreviations: APC, antigen-presenting cell; CAR, chimeric antigen receptor; CTLA-4, cytotoxic T-lymphocyte-associated protein 4; hTERT, human telomerase reverse transcriptase; ICI, immune checkpoint inhibitor; NK, natural killer; PAP, prostatic acid phosphatase; PD-1, programmed cell death protein 1; PD-L1, programmed death-ligand 1; PSA, prostate-specific antigen; Th1, T-helper type 1.

The biological rationale for immunotherapy in urological cancers is rooted in the distinct immunobiology of each tumor type, as detailed above. **Figure 1** provides a schematic synthesis of the principal immunotherapeutic classes, their biological targets, and the primary immune effectors engaged across bladder, kidney, and prostate malignancies, from checkpoint inhibition and BCG-induced local inflammation to cancer vaccines and adoptive cellular engineering.

### 1.3 Emerging drug delivery systems for immunotherapy

Beyond the agents themselves, advanced drug delivery systems are being developed to enhance immunotherapy efficacy. Nanocarriers for targeted ICI delivery (e.g., PD-1/PD-L1 antibody-loaded nanoparticles) have shown preclinical promise in bladder cancer models, improving tumor accumulation and reducing systemic immune-related adverse events. Biomaterial scaffolds for localized immune activation (e.g., implantable scaffolds releasing ICIs and cytokines) are being evaluated in prostate cancer to overcome the immunosuppressive tumor microenvironment. While these technologies have not yet reached phase III RCTs in urological cancers, they represent an important frontier (15, 16).

### 1.4 Challenges and opportunities

Despite these advances, challenges remain. Many patients do not respond to immunotherapy, and those who do may develop resistance. The optimal sequencing of therapies, the role of combination strategies, and the identification of predictive biomarkers remain active areas of investigation. Furthermore, the high costs of novel immunotherapies raise questions about equitable access and cost-effectiveness. Immune-related adverse events (irAEs), which can affect any organ system, require specialized management and can be severe or fatal.

This systematic review aims to synthesize the evidence from phase II/III randomized controlled trials, evaluating immunotherapy (ICIs, ADCs, vaccines, cellular therapies) in urological cancers (bladder, kidney, and prostate) published between 2010 and 2026. We describe how these trials have established new standards of care and identify emerging paradigms for patient management, while excluding hormonal monotherapy and targeted therapy without an immunotherapy component. Beyond conventional trial data, this review also examines the emerging synergy between immunotherapy and radiotherapy, alongside real-world evidence that complements RCT findings by illuminating treatment patterns, access disparities, and outcomes in unselected populations.

## 2 Methods

### 2.1 Protocol and registration

This systematic review was designed and conducted in accordance with the Preferred Reporting Items for Systematic Reviews and Meta-Analyses (PRISMA) 2020 statement. The protocol was not prospectively registered. The protocol was not prospectively registered in PROSPERO due to the retrospective nature of this work. The authors acknowledge this as a limitation. The PRISMA 2020 checklist is provided in Supplementary File 1.

### 2.2 Eligibility criteria

Inclusion criteria were: (1) phase II or III randomized controlled trials (RCTs) or single-arm phase II trials with practice-changing implications; (2) adult patients (≥18 years) with histologically confirmed bladder, kidney, or prostate cancer; (3) evaluation of an immunotherapeutic intervention, including ICIs, ADCs, cancer vaccines, adoptive cellular therapies, or immunomodulatory agents; (4) trials evaluating radiotherapy (external beam radiotherapy, stereotactic body radiotherapy, or brachytherapy) in combination with immunotherapy, either concurrently or sequentially, were included when reporting survival or response outcomes; (5) real-world evidence studies (prospective or retrospective observational cohorts, registry analyses, or database studies) reporting survival, response, or safety outcomes with immunotherapy in urological cancers were included to complement RCT findings; (6) reporting of overall survival (OS), progression-free survival (PFS), objective response rate (ORR), or treatment-related adverse events as primary or secondary endpoints; (7) publication in English between January 1, 2010, and May 1, 2026. To ensure feasibility while maintaining methodological rigor, we prioritized phase III RCTs; phase II trials and real-world studies were included only when they reported practice-changing findings or introduced novel mechanistic insights.

Exclusion criteria were: trials evaluating hormonal therapy alone (e.g., enzalutamide, abiraterone, apalutamide) without an immunotherapy component; radioligand therapy (e.g., [¹^77^Lu]Lu-PSMA-617); targeted therapy alone without ICI combination; and studies not reporting survival outcomes.

### 2.3 Information sources and search strategy

A systematic search of PubMed/MEDLINE and Embase was performed from January 1, 2010, to May 1, 2026. The search strategy combined Medical Subject Headings (MeSH) terms and free-text terms for immunotherapy (including ICIs, ADCs, cancer vaccines, and interleukin-2), urological malignancies (bladder, kidney, and prostate neoplasms), and publication types (phase II and III randomized controlled trials). Additional search terms were incorporated to capture immunotherapy-radiotherapy combinations (radiotherapy, stereotactic body radiotherapy, brachytherapy, chemoradiotherapy, and radiation) and real-world evidence (observational studies, registries, cohorts, retrospective analyses, population-based studies, and real-world data or evidence). The search was limited to English language, human studies, and publications from January 1, 2010, to May 1, 2026. Reference lists of included articles and relevant review articles were hand-searched for additional studies.

### 2.4 Study selection and data extraction

Two reviewers (N. Ismaili and S. El Majjaoui) independently screened titles/abstracts and then full texts against the eligibility criteria. The second reviewer screened 20% of randomly selected records; disagreements were resolved by consensus. Data were extracted using a standardized form including: study characteristics (phase, design, setting, NCT number), patient population, intervention and comparator, primary and secondary endpoints, results (HR, 95% CI, p-values), safety data (grade ≥3 adverse events, treatment-related deaths), and biomarker analyses.

### 2.5 Data synthesis

Given the substantial heterogeneity across tumor types (bladder, kidney, prostate), disease stages (localized to metastatic), and intervention classes (ICIs, ADCs, vaccines, cellular therapies), a formal meta-analysis was not feasible. Therefore, we performed a narrative synthesis with structured reporting by tumor type and disease stage, following the Synthesis Without Meta-analysis (SWiM) reporting guidelines.

Risk of bias assessment: The Cochrane Risk of Bias 2 (RoB2) tool was used to assess the landmark trials identified as practice-changing. For domain-level judgments, each trial was rated as “low risk, “ “some concerns, “ or “high risk” for the following domains: randomization process, deviations from intended interventions, missing outcome data, measurement of the outcome, and selection of reported results.

## 3 Results

### 3.1 Study selection

**Figure 2:**
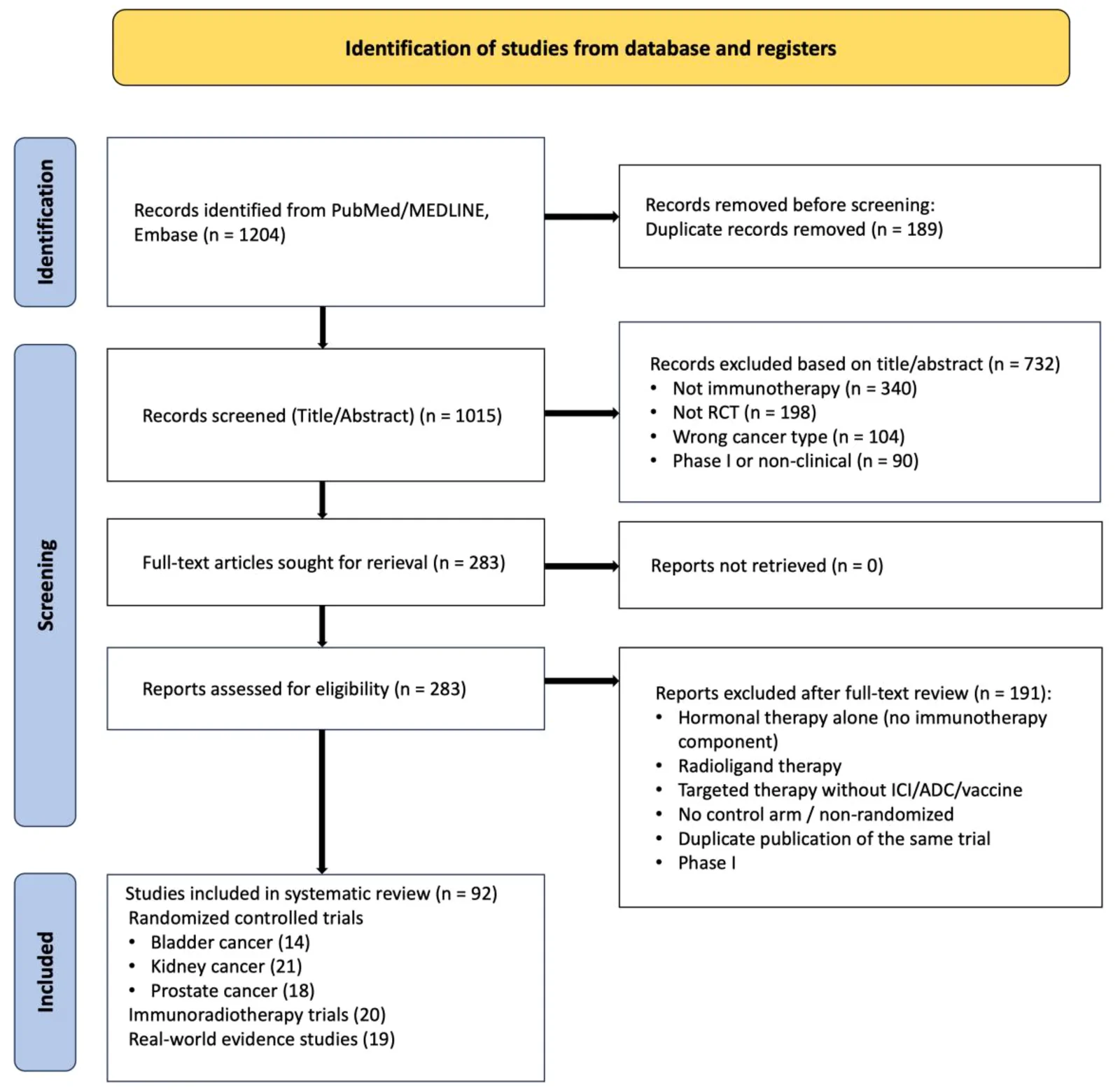
PRISMA 2020 flow diagram.

The initial search yielded 1204 records. After removing duplicates (n=189), 1015 records were screened by title and abstract, of which 732 were excluded (340 not immunotherapy, 198 not RCT, 104 wrong cancer type, 90 phase I or non-clinical). The remaining 283 reports were retrieved in full text and assessed for eligibility. Of these, 191 reports were excluded for the following reasons: hormonal therapy alone (n=59), radioligand therapy (n=26), targeted therapy without an immunotherapy component (n=42), no control arm or non-randomized design (n=17), duplicate publication of the same trial (n=30, including 13 duplicate references), and phase I only (n=17). Consequently, 92 studies met the inclusion criteria, comprising 53 clinical trials (bladder cancer: n=14; kidney cancer: n=21; prostate cancer: n=18), 20 immunoradiotherapy combination trials, and 19 real-world evidence studies (**Figure 2**).

### 3.2 Bladder cancer

#### 3.2.1 Non-muscle-invasive bladder cancer

##### 3.2.1.1 ICI + BCG combinations

The use of intravesical BCG immunotherapy has been the cornerstone of high-risk NMIBC management for decades, yet up to 40-50% of patients eventually experience recurrence or progression. The biological rationale for combining BCG with ICIs stems from the observation that BCG induces a robust local inflammatory response characterized by t-helper 1 cells (Th1) polarization, recruitment of CD4+ and CD8+ T cells, and upregulation of PD-L1 expression on tumor-infiltrating immune cells and bladder cancer cells themselves. This BCG-induced PD-L1 upregulation represents an adaptive resistance mechanism that may limit the durability of BCG responses, providing a compelling rationale for dual blockade of the PD-1/PD-L1 pathway. The question of whether adding a PD-L1 inhibitor to BCG can enhance antitumor immunity while mitigating resistance has been addressed by two large phase III trials with divergent results, underscoring the complexity of this combination approach (3).

Two phase III trials tested adding a PD-L1 inhibitor to BCG in BCG-naïve high-risk NMIBC. The POTOMAC trial investigated whether adding durvalumab to BCG could improve outcomes in BCG-naive, high-risk NMIBC. This phase III RCT enrolled 1018 patients who underwent transurethral resection of bladder tumor (TURBT) and were randomized 1:1:1 to receive: (1) durvalumab plus BCG induction and maintenance (2 years of BCG), (2) durvalumab plus BCG induction only, or (3) BCG induction and maintenance alone. The primary endpoint was investigator-assessed disease-free survival (DFS). At a median follow-up of 60.7 months, the durvalumab plus BCG induction and maintenance group showed a 32% reduction in the risk of recurrence or death compared with BCG alone (HR 0.68; 95% CI 0.50-0.93; p=0.015). The 24-month DFS was 86.5% versus 81.6%, respectively. The safety profile was manageable, with grade 3-4 treatment-related adverse events (TRAE) occurring in 21% of patients in the combination group versus 4% in the BCG-alone group. Immune-mediated adverse events occurred in 27% of patients receiving durvalumab, most commonly hypothyroidism. The trial demonstrated that one year of durvalumab combined with BCG induction and maintenance therapy represents a potential new treatment option for BCG-naive, high-risk NMIBC (17).

In contrast to POTOMAC, the phase III ALBAN trial comparing atezolizumab plus BCG versus BCG alone in BCG-naive, high-risk NMIBC showed no event-free survival (EFS) benefit with atezolizumab addition. This international, open-label, randomized trial enrolled 517 patients who received either BCG alone or BCG plus intravenous atezolizumab (1200 mg every 3 weeks for 1 year), followed by a 1-year BCG maintenance schedule. The primary endpoint of investigator-assessed EFS (including low-grade and high-grade recurrence, progression to ≥T2, metastasis, or death) was not met (HR 0.98; 95% CI 0.71-1.36; p=0.9106). EFS results were consistent across all prespecified subgroups, including patients with carcinoma *in situ* and T1 disease. TRAEs of grade ≥3 occurred in 22.7% of patients in the combination arm versus 8.8% in the BCG-alone arm. The divergent results between POTOMAC and ALBAN suggest that the benefit of PD-1/PD-L1 blockade in NMIBC may be agent-specific rather than a class effect, potentially related to differences in PD-L1 binding affinity, Fc effector function, or the specific BCG strain used (18).

##### 3.2.1.2 Vaccine priming

The phase I RUTIVAC-1 double-blind, placebo-controlled trial used the RUTI vaccine (derived from *Mycobacterium tuberculosis*) as a systemic primer before intravesical BCG in patients with high-risk NMIBC. The rationale was that prior exposure to mycobacterial antigens might enhance BCG-induced antitumor immunity. RUTI was safe and induced systemic vaccine-specific immune responses, with exploratory analyses showing trends toward reduced recurrence and progression. While not powered for efficacy, this trial established the feasibility of heterologous prime-boost strategies in NMIBC (19).

#### 3.2.2 Muscle-invasive bladder cancer

##### 3.2.2.1 Perioperative ICI and ADC combinations

The perioperative period represents a unique opportunity to harness immunotherapy for MIBC, to eliminate micrometastatic disease, and improve cure rates. The high TMB characteristic of urothelial carcinoma, second only to melanoma and lung cancer, generates numerous neoantigens that can be recognized by the immune system, making MIBC particularly amenable to immune checkpoint inhibition. Neoadjuvant immunotherapy may prime the immune system before surgical resection of the primary tumor, potentially inducing systemic antitumor immunity that persists after surgery. Furthermore, combining immunotherapy with chemotherapy may produce synergistic effects: chemotherapy can induce immunogenic cell death, release tumor antigens, and deplete immunosuppressive cell populations, while checkpoint inhibitors prevent T-cell exhaustion. This synergy has now been validated in several practice-changing trials, which have reshaped the curative-intent landscape for both cisplatin-eligible and cisplatin-ineligible patients (2, 20).

In the landmark NIAGARA phase III trial, perioperative durvalumab combined with neoadjuvant gemcitabine-cisplatin (GC) redefined the standard of care for cisplatin-eligible MIBC. Enrolling 1063 patients with MIBC (clinical stage T2-T4a, N0-N1, M0) who were eligible for cisplatin-based chemotherapy and medically fit for radical cystectomy (RC). Patients were randomized 1:1 to receive neoadjuvant durvalumab (1500 mg) plus GC every 3 weeks for four cycles, followed by RC and adjuvant durvalumab every 4 weeks for eight cycles (durvalumab group), or neoadjuvant GC followed by RC alone. With a median follow-up of 25.6 months, the durvalumab group showed a significant improvement in EFS (HR 0.68; 95% CI 0.56-0.82; p<0.0001), with 24-month EFS of 67.8% versus 59.8%. OS also favored durvalumab (HR 0.75; 95% CI 0.59-0.93; p=0.0106), with 24-month OS of 82.2% versus 75.2%. The pCR rate was not significantly different in the primary analysis (33.8% vs. 25.8%; p=0.004), though an updated analysis including previously omitted samples showed 37.3% vs. 27.5%. RC was performed in 88.0% of durvalumab group patients versus 83.2% in the comparison group. The safety profile was consistent with that of the individual agents. Although FDA approval followed, the trial design does not disentangle the contributions of the neoadjuvant versus adjuvant phases, a caveat that warrants ongoing investigation (21).

For the substantial subset of patients ineligible for or unwilling to receive cisplatin, the KEYNOTE-905/EV-303 trial provided a transformative, chemotherapy-free alternative. This phase III trial randomized 344 participants with cisplatin-ineligible or cisplatin-declining MIBC (T2-T4aN0M0 or T1-T4aN1M0) 1:1 to receive perioperative enfortumab vedotin (1.25 mg/kg on days 1 and 8) plus pembrolizumab (200 mg on day 1 every 3 weeks) followed by RC, or surgery alone. In the experimental arm (EV+pembrolizumab), patients received 3 cycles of NAT and, after surgery, 6 additional cycles of EV and 14 cycles of pembrolizumab. At a median follow-up of 25.6 months, the combination dramatically improved EFS (HR 0.40; 95% CI 0.28-0.57; p<0.001), with 2-year EFS of 74.7% versus 39.4%. Overall survival also favored the experimental arm (HR 0.50; 95% CI 0.33-0.74; p<0.001), with 2-year OS of 79.7% versus 63.1%. A pCR was achieved in 57.1% of patients receiving perioperative EV+pembrolizumab versus 8.6% of those undergoing surgery alone (p<0.001). Grade ≥3 TRAEs occurred in 45.5% of the combination group, compared with grade ≥3 any-cause adverse events in 45.9% of the control group (reflecting the morbidity of cystectomy alone in this frail population). Surgery was performed in 87.6% and 89.7% of patients, respectively, indicating that neoadjuvant therapy (NAT) did not compromise surgical feasibility (22).

Complementing these phase III data, the phase II AURA trial explored avelumab-based neoadjuvant strategies across cisplatin-eligibility strata. In cisplatin-eligible patients (n=79), avelumab combined with either ddMVAC or GC yielded pCR rates of 58% and 53%, respectively, with corresponding 36-month OS of 87% versus 67%. In the cisplatin-ineligible cohort (n=58), avelumab monotherapy unexpectedly outperformed its combination with paclitaxel-gemcitabine (pCR 32% vs 14%; 36-month OS 42% vs 48%), highlighting a potential role for single-agent immunotherapy in this setting. Collectively, these results support ddMVAC as a promising chemotherapy partner for neoadjuvant chemo-immunotherapy combinations in future phase III trials (23).

##### 3.2.2.2 ctDNA-guided adjuvant therapy

IMvigor011 trial investiged ctDNA-guided adjuvant atezolizumab in MIBC. This phase III RCT enrolled 761 patients with MIBC (ypT2-4aN0M0 or ypT0-4aN+M0) who were disease-free by imaging 6-24 weeks after RC. Patients underwent serial ctDNA monitoring (every 6 weeks for 9 months, then at 1 year) using a tumor-informed assay. Those who tested ctDNA-positive remained disease-free by imaging were randomized 2:1 to atezolizumab (1680 mg every 4 weeks) or placebo for up to 1 year. At a median follow-up of 16.1 months, median DFS was 9.9 months with atezolizumab versus 4.8 months with placebo (HR 0.64; 95% CI 0.47-0.87; p=0.005). Median OS was 32.8 months versus 21.1 months (HR 0.59; 95% CI 0.39-0.90; p=0.01). Among 357 patients with persistent ctDNA-negative status, DFS was 95% at 1 year and 88% at 2 years without adjuvant therapy. This trial demonstrated that ctDNA-guided adjuvant therapy can identify patients who benefit from immunotherapy while sparing low-risk patients from unnecessary treatment, representing a paradigm shift in precision adjuvant therapy (24).

##### 3.2.2.3 Novel mechanisms (GDF-15 blockade)

The phase II GDFather-NEO trial added visugromab (anti-GDF-15) to neoadjuvant nivolumab in MIBC. The combination tripled the pCR rate (33.3% vs 7.1%; major pathologic response 66.7% vs 21.4%) with no new safety signals, providing proof-of-concept for targeting the GDF-15 resistance pathway (25).

#### 3.2.3 Advanced/metastatic urothelial carcinoma

Locally-advanced or metastatic urothelial carcinoma (la/mUC) has long been associated with poor outcomes, with a median OS of approximately 12–15 months with platinum-based chemotherapy alone (26). The immunological vulnerability of this tumor type, driven by its exceptionally high mutational burden and consequent neoantigen load, provided the initial rationale for exploring checkpoint inhibitors in the metastatic setting. However, early trials of single-agent PD-1/PD-L1 inhibitors yielded response rates of only 15-25%, suggesting that additional mechanisms of immune evasion operate in advanced disease. ADCs such as EV and disitamab vedotin (DV) offer a complementary approach: beyond their direct cytotoxic effects, ADCs can induce immunogenic cell death, promote antigen release, and modulate the tumor microenvironment to enhance checkpoint inhibitor activity. This synergistic rationale, combining targeted cytotoxicity with immune checkpoint blockade, has now been validated in multiple phase III trials, establishing ADC-ICI combinations as a new standard of care for first-line metastatic disease (26, 27).

##### 3.2.3.1 First-line standard: ADC+ICI

The EV-302/KEYNOTE-A39 phase III RCT established EV plus pembrolizumab as a standard of care for first-line treatment of la/mUC, irrespective of cisplatin eligibility or PD-L1 expression. The trial enrolled 886 previously untreated patients with measurable disease per RECIST v1.1, and adequate organ function (including GFR ≥30 ml/min). Patients were randomized 1:1 to receive EV+pembrolizumab every 3 weeks, or platinum-based chemotherapy (GC) on the same schedule. At a median follow-up of 29.1 months (2.5 years), the ADC-ICI combination significantly reduced the risk of progression or death by 52% (HR 0.48; 95% CI 0.41–0.57; nominal p<0.00001) and the risk of death by 49% (HR 0.51; 95% CI 0.43–0.61; nominal p<0.00001). Median PFS was 12.5 versus 6.3 months, and median OS was 33.8 versus 15.9 months, respectively. The confirmed ORR was 67.5% versus 44.2%, with a median duration of response of 23.3 months (vs. 7.0 months). Among patients achieving a CR, the probability of remaining in CR at 24 months was 74.3% with EV+pembrolizumab versus 43.2% with chemotherapy. Grade ≥3 TRAEs occurred in 57.3% of patients in the experimental arm versus 69.5% in the chemotherapy arm, with no new safety signals observed after extended follow-up. This trial definitively established EV+pembrolizumab as the preferred first-line regimen for la/mUC, transforming the treatment landscape for all patients regardless of cisplatin eligibility or PD-L1 status (28).

For patients with HER2-expressing tumours (IHC 1+, 2+, 3+), the phase III RC48-C016 trial compared disitamab vedotin (2.0 mg/kg) plus toripalimab (anti-PD-1 antibody) (3 mg/kg) every 2 weeks vs platinum-based chemotherapy (GC) every 3 weeks as first-line treatment. A total of 484 patients were randomized 1:1. At a median follow-up of 18.2 months, the DV+toripalimab group demonstrated significantly longer PFS (median 13.1 vs. 6.5 months; HR 0.36; 95% CI 0.28–0.46; p<0.001) and OS (median 31.5 vs. 16.9 months; HR 0.54; 95% CI 0.41–0.73; p<0.001) compared with chemotherapy. The ORR was 76.1% versus 50.2%, and the median duration of response was 14.6 versus 5.6 months. The safety profile favored the experimental regimen, with grade ≥3 treatment-related adverse events occurring in 55.1% of patients receiving DV plus toripalimab versus 86.9% receiving chemotherapy. This trial established a new biomarker-driven first-line treatment option for patients with HER2-expressing la/mUC, representing a new paradigm in precision immuno-oncology for bladder cancer (29).

Finally, the CheckMate 901 trial offered an alternative for cisplatin-ineligible patients: nivolumab plus ipilimumab improved OS vs gemcitabine-carboplatin (HR 0.79; p=0.0245), providing a chemotherapy-free option (30).

##### 3.2.3.2 Maintenance and de-escalation strategies

The JAVELIN Bladder 100 trial III RCT established avelumab as first-line maintenance as standard of care for la/mUC without progression after platinum-based chemotherapy. Eligible patients had unresectable la/mUC, had not progressed after 4-6 cycles of first-line platinum-based chemotherapy (cisplatin or carboplatin plus gemcitabine), and had ECOG PS 0-1. After a 4-10 week treatment-free interval, 700 patients were randomized 1:1 to avelumab (10 mg/kg every 2 weeks) plus best supportive care (BSC) or BSC alone. At the primary analysis, median OS, was 21.4 months with avelumab vs. 14.3 months with BSC (HR 0.69; 95% CI 0.56-0.86; p=0.001). Long-term analyses with ≥2 years of follow-up confirmed sustained OS benefit (median 23.8 vs. 15.0 months; HR 0.76; 95% CI 0.63-0.91; p=0.0036). Subgroup analyses showed that patients with nonvisceral metastases (including bone) or lymph node-only disease derived particular benefit, with median OS of 31.4 versus 17.1 months (HR 0.60) and 31.9 versus 22.7 months (HR 0.86), respectively. Older patients (≥65 years, n=464, 66.3% of the population) also derived benefit, with median OS of 26.1 vs. 15.5 months (HR 0.70). Patient-reported outcomes and quality-adjusted time without symptoms or toxicity (Q-TWiST) analyses showed no detrimental impact on quality of life (QOL), with a 4.57-month gain in Q-TWiST favoring avelumab (30.35% relative improvement). Safety was consistent with the known profile of avelumab, with grade ≥3 treatment-related adverse events in 19.5% of older patients (31, 32).

Conversely, the phase II DISCUS trial compared three vs six cycles of platinum-based chemotherapy, both followed by avelumab maintenance. Three cycles significantly improved QOL (+8.5 points; p=0.016) with identical OS (18.9 months both arms) and fewer grade ≥3 TRAEs (6% vs 11%), supporting abbreviated chemotherapy as a patient-centred option (33).

##### 3.2.3.3 Upper tract urothelial carcinoma

The phase II DISTINCT-I trial evaluated a kidney-sparing strategy with DV plus tislelizumab followed by endoscopic or segmental resection in high-risk UTUC with imperative renal preservation. The 1-year kidney-intact EFS was 70%, with a clinical CR rate of 75% after 4 months and no grade ≥3 systemic toxicities. This challenges the historical standard of radical nephroureterectomy (34).

The results of the 14 key clinical trials in bladder cancer are summarized in **Table 1**.

**Table:** Table 1 Practice-changing randomized trials in bladder cancer: from perioperative intensification to precision immunotherapy.

Trial Name/reference	Phase	Population	Experimental arm	Control arm	Primary endpoint(s)	Key efficacy results	Key safety/tolerability results
POTOMAC (17)	III	BCG-naïve, high-risk NMIBC	Durvalumab + BCG (induction + maintenance)	BCG alone	DFS	HR 0.68; 24-mo DFS 86.5% vs 81.6%; p=0.015	Grade 3-4 TRAEs 21% vs 4%; immune-mediated AEs 27%
ALBAN (18)	III	BCG-naïve, high-risk NMIBC	Atezolizumab + BCG (1 year)	BCG alone	EFS	HR 0.98; p=0.91 (negative)	Grade ≥3 TRAEs 22.7% vs 8.8%
RUTIVAC-1 (19)	I/II	High-risk NMIBC	RUTI vaccine (prime) + BCG	Placebo + BCG	Immunogenicity	Safe; induced systemic vaccine-specific responses	Not reported
NIAGARA (21)	III	Cisplatin-eligible MIBC	Perioperative durvalumab + GC	Neoadjuvant GC alone	EFS/pCR	EFS HR 0.68; OS HR 0.75; pCR 37.3% vs 27.5%	Consistent with individual agents
KEYNOTE-905/EV-303 (22)	III	Cisplatin-ineligible/declining MIBC	Perioperative EV + pembrolizumab	Surgery alone	EFS	EFS HR 0.40; OS HR 0.50; pCR 57.1% vs 8.6%	Grade ≥3 TRAEs 45.5%; surgery 87.6% vs 89.7%
AURA (23)	II	MIBC (cisplatin-eligible and -ineligible)	Avelumab + ddMVAC or GC (eligible); avelumab ± PG (ineligible)	Randomised comparison	pCR	Eligible: pCR 58% vs 53%; Ineligible: pCR 32% vs 14%	Not reported
IMvigor011 (24)	III	ctDNA+ MIBC post-cystectomy	Adjuvant atezolizumab	Placebo	DFS/OS	DFS HR 0.64; OS HR 0.59	Consistent with atezolizumab profile
GDFather-NEO (25)	II	MIBC	Neoadjuvant nivolumab + visugromab	Placebo + nivolumab	pCR	pCR 33.3% vs 7.1%; MPR 66.7% vs 21.4%	No new safety signals
EV-302/KEYNOTE-A39 (28)	III	First-line la/mUC	EV + pembrolizumab	Platinum-based chemotherapy	PFS/OS	PFS HR 0.48; OS HR 0.51; ORR 67.5% vs 44.2%	Grade ≥3 TRAEs 57.3% vs 69.5%
RC48-C016 (29)	III	First-line HER2+ la/mUC	Disitamab vedotin + toripalimab	Platinum-based chemotherapy	PFS/OS	PFS HR 0.36; OS HR 0.54; ORR 76.1% vs 50.2%	Grade ≥3 TRAEs 55.1% vs 86.9%
CheckMate 901 (30)	III	Cisplatin-ineligible la/mUC	Nivolumab + ipilimumab	Gemcitabine-carboplatin	OS	OS HR 0.79; p=0.0245	Not reported
JAVELIN Bladder 100 (31, 32)	III	la/mUC without progression after platinum	Avelumab maintenance + BSC	BSC alone	OS	OS HR 0.76; median OS 23.8 vs 15.0 mo	Grade ≥3 TRAEs 19.5% (older patients)
DISCUS (33)	II	First-line la/mUC	3 cycles platinum + avelumab maintenance	6 cycles platinum + avelumab maintenance	PFS/QoL	Identical OS (18.9 mo); QoL improved with 3 cycles	Grade ≥3 TRAEs 6% vs 11%
DISTINCT-I (34)	II	High-risk upper tract UC	Disitamab vedotin + tislelizumab (nephron-sparing)	—	pCR	1-yr kidney-intact EFS 70%; clinical CR 75%	No grade ≥3 systemic toxicities

AE, adverse event; BSC, best supportive care; CI, confidence interval; CR, complete response; ctDNA, circulating tumour DNA; ddMVAC, dose-dense methotrexate-vinblastine-doxorubicin-cisplatin; DFS, disease-free survival; DLT, dose-limiting toxicity; EFS, event-free survival; EV, enfortumab vedotin; GC, gemcitabine-cisplatin; HER2, human epidermal growth factor receptor 2; HR, hazard ratio; la/mUC, locally advanced/metastatic urothelial carcinoma; MIBC, muscle-invasive bladder cancer; mo, months; MPR, major pathologic response; NMIBC, non-muscle-invasive bladder cancer; NR, not reported; ORR, objective response rate; OS, overall survival; pCR, pathological complete response; PD-1, programmed cell death protein 1; PD-L1, programmed death-ligand 1; PFS, progression-free survival; PG, paclitaxel-gemcitabine; QoL, quality of life; SBRT, stereotactic body radiotherapy; TRAE, treatment-related adverse event; UC, urothelial carcinoma.

### 3.3 Kidney cancer

#### 3.3.1 Localized disease (adjuvant and neoadjuvant)

Patients with high-risk RCC face recurrence rates of 40–50% within five years of nephrectomy, underscoring the urgent need for effective adjuvant strategies. The intrinsic immunogenicity of clear cell RCC, evidenced by spontaneous regressions and durable responses to cytokines, provides a strong biological foundation for checkpoint inhibition in the minimal-residual-disease setting. By targeting occult micrometastases when tumour burden is low, adjuvant immunotherapy offers the potential to eradicate residual clones before they establish clinically overt metastases. Yet, as the following trials illustrate, success is highly agent- and biomarker-dependent, with contrasting outcomes that have refined our understanding of optimal patient selection (35).

The definitive advance in this space came with the KEYNOTE-564 trial, which established pembrolizumab as the reference standard for adjuvant therapy. In this phase III study, 994 patients with intermediate-high risk (pT2 with grade 4 or sarcomatoid differentiation, N0, M0; or pT3 any grade, N0, M0), high risk (pT4 any grade, N0, M0; or any pT, N+, M0), or M1 with no evidence of disease after complete resection of oligometastases, were randomized 1:1 to pembrolizumab or placebo for up to 17 cycles. With 30 months of follow-up, the DFS HR was 0.63 (95% CI 0.50-0.80), with 30-month DFS rates of 75.2% vs. 65.5%. The OS HR was 0.52 (95% CI 0.31-0.86). Subgroup analyses showed consistent benefit regardless of PD-L1 status, sarcomatoid features, or M1 NED status. Patient-reported outcomes showed no clinically meaningful differences between arms, with most scores remaining stable or improved. Grade ≥3 adverse events occurred in 32% vs. 18%, with immune-mediated adverse events in 36% vs. 7%. The East Asian subgroup analysis showed consistent DFS (HR 0.70) and OS (HR 0.47) benefits (36, 37).

Building on the concept of intensified checkpoint blockade, the RAMPART phase III trial evaluated whether combining durvalumab (1500 mg every 4 weeks for up to 1 year) with tremelimumab (300 mg × 1 dose or 75 mg × 4 doses) could further improve outcomes over active monitoring in high-risk resected RCC. The dual ICI combination significantly improved DFS versus active monitoring (HR 0.65; 95% CI 0.53-0.80; p<0.001), expanding the adjuvant armamentarium beyond pembrolizumab monotherapy. However, this intensified regimen came at a cost, with grade 3–4 adverse events occurring in 38% of treated patients, a trade-off that must be weighed against the incremental benefit (38).

In stark contrast, the IMmotion010 trial failed to meet its primary DFS endpoint for adjuvant atezolizumab versus placebo (HR 0.93; p=0.50) in a similarly high-risk population. This negative result, however, yielded a landmark biomarker discovery. In this phase III RCT, 778 patients with increased risk of recurrence (pT2 or higher, or any pT with nodal involvement, or M1 NED), were randomized 1:1 to atezolizumab (1200 mg every 3 weeks for 16 cycles or 1 year) or placebo. The exploratory biomarker analyses results identified plasma kidney injury molecule-1 (KIM-1) as a potential predictive biomarker. KIM-1 is a transmembrane glycoprotein overexpressed in clear cell and papillary RCC, whose extracellular domain can be detected in serum. Using a high-throughput proteomics assay (Olink Explore 3072/384), KIM-1 was identified as the most significantly enriched protein at recurrence versus baseline. Patients with high baseline serum KIM-1 (≥86 pg/mL, 60th percentile) had worse prognosis (DFS HR 1.68; 95% CI 1.35-2.09) but derived significant benefit from adjuvant atezolizumab (DFS HR 0.72; 95% CI 0.52-0.99), while those with low KIM-1 did not (HR 1.20; 95% CI 0.88-1.63). An increase in KIM-1 during follow-up was associated with worse DFS, particularly in KIM-1-high patients treated with placebo (median DFS 4.8 months) compared with atezolizumab (14.8 months). These findings position KIM-1 as a promising circulating biomarker for minimal residual disease, paving the way for biomarker-driven adjuvant paradigms, pending prospective validation (39).

The perioperative strategy, integrating immunotherapy both before (1-4 weeks) and after surgery, was tested in the PROSPER trial, where a single preoperative nivolumab dose (240 mg) followed by nine months of postoperative treatment failed to improve recurrence-free or OS versus surgery alone in patients with high-risk RCC. Despite this negative result, the trial proved highly valuable for prospectively validating the pragmatic GRANT (GRade, Age, Nodes, Tumor) score. Among 714 evaluable patients, the GRANT score (one point each for age >60 years, grade >2, pT ≥pT3b, and pN1) effectively stratified prognosis into favourable (0-1 factors) and unfavourable (2-4 factors) groups, with markedly different RFS (61.1 vs 36.9 months; HR 0.36) and OS (HR 0.25). While the score did not predict immunotherapy benefit (no treatment-by-score interaction), its strong prognostic performance, particularly in non-clear cell RCC (c-index 0.74), supports its integration into routine risk stratification (40).

Complementing these larger adjuvant efforts, the phase II NESCIO trial explored short-course neoadjuvant regimens in high-risk resectable clear cell RCC. Pathologic response rates remained modest across all arms (7–14%), but the ipilimumab-containing doublet was associated with a 42.8% rate of grade ≥3 immune-related adverse events. These findings provide critical context for the risk-benefit calculus of neoadjuvant immunotherapy, cautioning against routine use outside of well-controlled trials (41).

#### 3.3.2 Metastatic clear cell RCC - first-line therapy

The distinct immunobiology of clear cell RCC, rooted in VHL inactivation, constitutive HIF activation, and the resulting crosstalk between angiogenesis and immune modulation, has long provided a compelling rationale for dual checkpoint blockade and VEGF–immune combinations. Five major phase III trials now furnish mature, practice-defining evidence for first-line regimens, while smaller phase II studies are refining biomarkers and exploring next-generation targets (2).

Long-term follow-up of checkMate 214 trial, a phase III RCT, established the combination of nivolumab plus ipilimumab as a first-line therapy for intermediate/poor-risk advanced RCC. A total of 1, 096 patients were randomized 1:1 to nivolumab (3 mg/kg every 3 weeks for 4 doses) plus ipilimumab (1 mg/kg every 3 weeks for 4 doses), followed by nivolumab maintenance (3 mg/kg every 2 weeks or 480 mg every 4 weeks), or sunitinib (50 mg daily, 4 weeks on/2 weeks off). At 5-year follow-up (minimum 60 months), median OS was 55.7 months with nivolumab plus ipilimumab versus 38.4 months with sunitinib in intermediate/poor-risk patients (HR 0.72; 95% CI 0.62-0.85). In favorable-risk patients, median OS was not reached in either arm (HR 0.94). Treatment-free survival analysis showed that intermediate/poor-risk patients treated with nivolumab plus ipilimumab had longer survival and longer TFS without toxicity versus sunitinib (10.1 vs. 4.1 months at 60 months). In patients with sarcomatoid features, nivolumab plus ipilimumab demonstrated remarkable efficacy with median OS of 48.6 vs. 14.2 months (HR 0.46) and ORR 60.8% vs. 23.1% (42–44).

Four subsequent phase III trials established ICI plus VEGFR-TKI as equally potent first-line alternatives, each with distinct efficacy profiles. CheckMate 9ER (nivolumab+cabozantinib) randomised 651 patients to nivolumab (240 mg every 2 weeks) plus cabozantinib (40 mg daily) versus sunitinib. At 5.6 years of follow-up, median PFS was 16.4 versus 8.3 months (HR 0.58), median OS 46.5 versus 35.5 months (HR 0.79), and ORR 55.7% versus 27.4% (CR 13.9% vs 4.6%). Subgroup analyses mirrored CheckMate 214 in showing no OS benefit for favourable-risk patients (HR 1.08), while partitioned survival analysis revealed that the survival advantage was driven by more time on first-line therapy (22.6 vs 14.1 months at 48 months) and in TFS (7.0 vs 4.6 months), with less time spent on subsequent therapies (5.5 vs 12.0 months). Grade ≥3 TRAEs occurred in 63% of patients (45–47).

The KEYNOTE-426 trial (pembrolizumab+axitinib) randomised 861 patients to pembrolizumab (200 mg every 3 weeks for up to 35 cycles) plus axitinib (5 mg twice daily) versus sunitinib. At 5-year follow-up (median 67.2 months), pembrolizumab plus axitinib showed sustained OS (HR 0.84; 95% CI 0.71-0.99), PFS (HR 0.69; 95% CI 0.59-0.81), and ORR (60.6% vs. 39.6%) benefits. Among patients who completed 35 cycles of pembrolizumab (n=120), the 48-month and 60-month OS rates were 81.7% and 70.7%, respectively. An 18-gene T-cell-inflamed gene expression profile (TcellinfGEP) was positively associated with ORR (p<0.0001), PFS (p<0.0001), and OS (p=0.002) within the pembrolizumab plus axitinib arm, but not within the sunitinib arm, supporting TcellinfGEP as a predictive biomarker for pembrolizumab response. Conversely, an angiogenesis signature was positively associated with outcomes within the sunitinib arm. PBRM1 mutation was associated with improved ORR in the pembrolizumab plus axitinib arm (71.4% vs. 52.3%; p=0.002). PD-L1 CPS was not associated with outcomes in the combination arm, suggesting it should not be used to select patients for pembrolizumab plus axitinib (48).

CLEAR phase III RCT (lenvatinib+pembrolizumab) randomised 1069 patients 1:1:1 to lenvatinib (20 mg daily) plus pembrolizumab (200 mg every 3 weeks), lenvatinib plus everolimus, or sunitinib. At final OS analysis with ~4 years of follow-up, lenvatinib+pembrolizumab demonstrated the most pronounced PFS benefit (HR 0.39; median PFS 23.9 vs 9.2 months), the highest ORR (71% vs 36%), and a 16.1% CR rate (vs 4.2% with sunitinib). Post-hoc analysis by baseline metastatic characteristics showed consistent PFS and ORR benefits regardless of metastatic site (lung: median PFS 22.1 vs. 6.0 months, HR 0.41; lymph node: 22.0 vs. 7.5 months, HR 0.49; bone: 17.2 vs. 5.6 months, HR 0.50; liver: 14.6 vs. 4.2 months, HR 0.48) or number of metastatic sites (one site: median PFS 32.2 vs. 13.8 months, HR 0.60; ≥2 sites: 20.8 vs. 5.6 months, HR 0.40). The probability of remaining in response through 60 months was 22.0% with lenvatinib plus pembrolizumab versus 10.0% with sunitinib. However, this efficacy comes at the cost of the highest grade ≥3 TRAE rate (72%), predominantly driven by lenvatinib-related hypertension (35%), diarrhoea (8%), and PPE (6%), necessitating careful dose management (49).

Finally, the JAVELIN Renal 101 trial (avelumab+axitinib) differs from the other trials by employing a PD-L1 inhibitor rather than a PD-1 inhibitor. This international, open-label, RCT enrolled 886 previously untreated patients to receive avelumab (10 mg/kg intravenously every 2 weeks) plus axitinib or sunitinib. The two independent primary endpoints were PFS and OS in patients with PD-L1-positive tumours (≥1% on immune cells, using the VENTANA PD-L1 [SP263] assay). Among 560 patients with PD-L1-positive tumours (63.2% of the population), median PFS was 13.8 months with avelumab plus axitinib versus 7.2 months with sunitinib (HR 0.61; 95% CI 0.47–0.79; p<0.001). In the overall population, median PFS was 13.8 versus 8.4 months (HR 0.69; 95% CI 0.56–0.84; p<0.001). The ORR in PD-L1-positive patients was 55.2% vs. 25.5%. However, the OS difference was not significant. Grade ≥3 TRAEs occurred in 71.2% of patients in the combination arm versus 71.5% in the sunitinib arm. The PD-L1-driven primary analysis and negative OS data have positioned this regimen slightly behind PD-1-based combinations in some clinical settings (50).

Beyond CTLA-4, alternative checkpoint targets are being evaluated. The phase II FRACTION-RCC RCT, compared nivolumab plus relatlimab (LAG-3 inhibitor) against nivolumab plus ipilimumab in IO-naive patients with advanced RCC. Thirty patients each were treated with nivolumab (240 mg) plus relatlimab (80 mg) every 2 weeks or nivolumab (3 mg/kg) plus ipilimumab (1 mg/kg) every 3 weeks for 4 doses, followed by nivolumab maintenance. ORR was 30% (95% CI 15-49%) with nivolumab plus relatlimab versus 20% (8-39%) with nivolumab plus ipilimumab. Median DOR was 33 weeks (16-53 weeks) versus not reached (33 weeks-NE). Grade 3-4 treatment-related adverse events occurred in 13% vs. 33%. Higher baseline LAG-3 and PD-L1 expression levels were detected among responders to nivolumab plus relatlimab. While the study was not designed to make direct comparisons between arms, these results suggested that LAG-3 inhibition may have antitumor activity with a more favorable safety profile than CTLA-4 blockade in RCC (51).

Conversely, attempts to de-escalate or resequence therapy have yielded largely negative results. The NIVOSWITCH phase II trial, which randomized patients with stable disease on first-line TKI to either continue TKI or switch to nivolumab maintenance, was prematurely terminated due to slow accrual. Patients with partial response or stable disease after 10-12 weeks of first-line TKI therapy (sunitinib or pazopanib) were randomized 1:1 to continue TKI or switch to nivolumab (240 mg every 2 weeks for 8 doses, then 480 mg every 4 weeks). The switch strategy proved clearly inferior, with worse ORR (20.0% vs 52.2%) and PFS (median 3.0 vs 11.9 months; HR 2.57), establishing that TKI continuation should remain the standard in responding patients (52).

Similarly, the MEDI0680 (anti-PD-1) plus durvalumab phase II trial showed no improvement in ORR (16.7% vs. 23.8%) or PFS (median 3.6 months in both arms) over nivolumab monotherapy, confirming that adding an anti-PD-1 antibody to an anti-PD-L1 antibody does not enhance efficacy in RCC, likely because single-agent PD-L1 blockade already achieves complete pathway inhibition (53).

Parallel efforts have focused on refining biomarker-driven selection. The IMmotion150 trial, though negative for its PFS endpoints, provided foundational insights by identifying three transcriptomic signatures, angiogenesis, T-effector/IFN-γ, and myeloid inflammation, that differentially associated with treatment benefit. High angiogenesis signature favoured sunitinib (PFS HR 0.31), while high T-effector signature favoured atezolizumab plus bevacizumab (HR 0.55 vs sunitinib); myeloid inflammation conferred resistance to atezolizumab monotherapy, which was overcome by bevacizumab (54). Building on this concept, the phase II OPTIC RCC trial prospectively used RNA-sequencing to classify patients into Angiogenic/Stromal or Immune clusters, assigning therapy accordingly (nivolumab+cabozantinib for Angiogenic/Stromal, achieving 71% ORR; ipilimumab+nivolumab for Immune clusters). This proof-of-concept moves beyond clinical risk stratification toward molecularly guided first-line selection (55).

Lastly, practical considerations for combination therapy were addressed by the CheckMate 800 trial, which compared fixed-ratio combination versus sequential infusion of nivolumab plus ipilimumab. Both methods proved safe with no clinically meaningful difference in anaphylactic reactions, providing practical guidance for administration (56).

#### 3.3.3 Metastatic RCC – second-line and post-immunotherapy

For patients with advanced clear-cell RCC progressing after one or two lines of antiangiogenic therapy, the phase III CheckMate 025 trial established nivolumab as the standard second-line therapy. This open-label, randomized trial enrolled 821 patients with previously treated advanced clear-cell RCC who had received one or two prior antiangiogenic regimens. Patients were randomized 1:1 to receive nivolumab 3 mg/kg intravenously every 2 weeks or everolimus 10 mg orally once daily. Median OS was significantly prolonged with nivolumab versus everolimus (25.0 vs 19.6 months; HR 0.73; 98.5% CI 0.57–0.93; p=0.002), meeting the prespecified superiority criterion. The ORR was substantially higher with nivolumab (25% vs 5%; odds ratio 5.98; p<0.001). No significant difference in PFS was observed (median 4.6 vs 4.4 months; HR 0.88; p=0.11). Grade 3–4 treatment-related adverse events were fewer with nivolumab than with everolimus (19% vs 37%), with fatigue (2%) being the most common grade 3 event with nivolumab and anemia (8%) with everolimus. This trial established nivolumab as a well-tolerated and effective second-line option for patients progressing after antiangiogenic therapy, setting the stage for subsequent ICI-TKI combinations in the first-line setting (57).

The FRUSICA-2 trial, a phase II RCT, established fruquintinib plus sintilimab as a highly effective second-line option after ICI progression in advanced RCC. Patients with advanced RCC who had progressed on or after ICI-based therapy were randomized to fruquintinib (a selective VEGFR-1/2/3 inhibitor) plus sintilimab (anti-PD-1 antibody) versus sunitinib. The primary endpoint was PFS, which was significantly improved with the combination (22.2 vs. 6.9 months; HR 0.373; 95% CI 0.255-0.545; p<0.0001). This trial addressed the important clinical question of optimal therapy after progression on frontline ICI combinations (58).

The CALYPSO trial evaluated durvalumab plus savolitinib (MET inhibitor) or tremelimumab in patients progressing on prior VEGF therapy. The durvalumab+tremelimumab arm showed a confirmed response rate of 33%, with no survival benefit in PD-L1-positive subgroups (59).

#### 3.3.4 Non-clear cell and special populations

##### 3.3.4.1 Papillary RCC

A retrospective analysis compared outcomes of nivolumab plus ipilimumab between patients with papillary RCC (n=14) and clear cell RCC (n=23). The ORR was 14.2% vs. 52.1% (p=0.03), and median PFS was 2.4 vs. 28.1 months (p<0.001). This highlighted the lower immunogenicity of papillary RCC and the need for alternative strategies (60).

##### 3.3.4.2 Sarcomatoid RCC

A prespecified subgroup analysis of the IMmotion010 trial evaluated adjuvant atezolizumab in patients with sarcomatoid RCC (sRCC). Among 104 patients with sRCC, the median DFS was not evaluable (95% CI 12 months-NE) with atezolizumab (n=37) versus 23 months (95% CI 11-NE) with placebo (n=67; HR 0.77; 95% CI 0.44-1.4). Grade 3-4 treatment-related adverse events occurred in 2.7% vs. 3.0%. While the difference was not statistically significant, the small sample size limits interpretation. The high baseline PD-L1 expression in sRCC (79% vs. 60% in ITT) suggests that this subgroup may be particularly immunogenic (61).

The key clinical studies included in this review for kidney cancer, covering adjuvant, perioperative stages, first-line strategies, maintenance switches and subsequent lines, are summarized in **Table 2**.

**Table:** Table 2 Landmark trials defining first-line therapy in renal cell carcinoma: dual checkpoint blockade and ICI-TKI combinations.

Trial Name/reference	Phase	Population	Experimental arm	Control arm	Primary endpoint(s)	Key efficacy Results	Key safety/tolerability results
KEYNOTE-564 (35–37)	III	Adjuvant, high-risk RCC post-nephrectomy	Pembrolizumab (up to 17 cycles)	Placebo	DFS	DFS HR 0.63; 30-mo DFS 75.2% vs 65.5%; OS HR 0.52	Grade ≥3 AEs 32% vs 18%; immune AEs 36% vs 7%
RAMPART (38)	III	Adjuvant, high-risk resected RCC	Durvalumab + tremelimumab	Active monitoring	DFS	DFS HR 0.65; p<0.001	Grade 3-4 AEs 38%
IMmotion010 (39)	III	Adjuvant RCC (pT2+ or N+ or M1 NED)	Atezolizumab	Placebo	DFS	DFS HR 0.93; p=0.50 (negative); KIM-1 predictive	Not reported
PROSPER (40)	III	Perioperative RCC	Nivolumab (1 dose pre-op + 9 mo post-op)	Observation	RFS/OS	Negative; GRANT score validated	Not reported
NESCIO (41)	II	Neoadjuvant, locally advanced ccRCC	Nivolumab + ipilimumab	—	pCR	pCR 7-14% (modest)	Grade ≥3 irAEs 42.8% (ipilimumab arm)
CheckMate 214 (42–44)	III	First-line intermediate/poor-risk aRCC	Nivolumab + ipilimumab	Sunitinib	OS	5-yr OS HR 0.72; median OS 55.7 vs 38.4 mo; sarcomatoid HR 0.46	TFS without toxicity 10.1 vs 4.1 mo at 60 mo
CheckMate 9ER (45–47)	III	First-line aRCC	Nivolumab + cabozantinib	Sunitinib	PFS/OS	PFS HR 0.58; OS HR 0.79; ORR 55.7% vs 27.4%	Grade 3-4: hypertension 13.1%, PPE 7.8%, diarrhea 7.2%
KEYNOTE-426 (48)	III	First-line aRCC	Pembrolizumab + axitinib	Sunitinib	OS	5-yr OS HR 0.84; PFS HR 0.69; ORR 60.6% vs 39.6%	TcellinfGEP predictive
CLEAR (49)	III	First-line aRCC	Lenvatinib + pembrolizumab	Sunitinib	PFS	PFS benefit across all metastatic sites (HR 0.39-0.60)	Not reported
JAVELIN Renal 101 (50)	III	First-line aRCC	Avelumab + axitinib	Sunitinib	PFS (PD-L1+)	PFS HR 0.61 (PD-L1+); overall PFS HR 0.69; ORR 55.2% vs 25.5%	Grade ≥3 TRAEs 71.2% vs 71.5%
FRACTION-RCC (51)	II	First-line IO-naïve aRCC	Nivolumab + relatlimab	Nivolumab + ipilimumab	ORR	ORR 30% vs 20%; DOR 33 wk vs NR	Grade 3-4 TRAEs 13% vs 33%
NIVOSWITCH (52)	II	Switch maintenance after TKI	Nivolumab	Continue TKI	OS (immature)	ORR 20.0% vs 52.2% (p=0.034); PFS HR 2.57 favouring TKI	Not reported
MEDI0680 + durvalumab (53)	II	Second-line (post-antiangiogenic)	MEDI0680 + durvalumab	Nivolumab	ORR	ORR 16.7% vs 23.8%; median PFS 3.6 mo both arms	Not reported
IMmotion1500 (54)	II	First-line mRCC	Atezolizumab ± bevacizumab	Sunitinib	PFS	Negative for PFS; biomarker insights (angiogenesis, T-effector)	Not reported
OPTIC RCC (55)	II	First-line ccRCC	Cabozantinib + nivolumab (Angiogenic/Stromal cluster)	—	PFS (by RNA cluster)	ORR 71% (Angiogenic/Stromal cluster)	Not reported
CheckMate 800 (56)	II	First-line aRCC (and melanoma)	Nivolumab + ipilimumab (fixed-ratio)	Sequential infusion	Safety	Both safe; no clinically relevant difference	Acceptable safety profiles
CheckMate 025 (57)	III	mRCC progressing after 1–2 antiangiogenic therapies	Nivolumab	Everolimus	OS	mOS 25.0 vs 19.6 mo (HR 0.73; p=0.002); ORR 25% vs 5% (p<0.001); PFS NS	Grade 3–4 TRAEs 19% vs 37%; most common: fatigue 2% (nivolumab), anemia 8% (everolimus)
FRUSICA-2 (41)	II	Second-line post-IO	Fruquintinib + sintilimab	Sunitinib	PFS	PFS HR 0.373; median PFS 22.2 vs 6.9 mo	Not reported

AE, adverse event; aRCC, advanced renal cell carcinoma; ccRCC, clear cell renal cell carcinoma; CI, confidence interval; DFS, disease-free survival; DOR, duration of response; GRANT, Grade, Age, Nodes, Tumor score; HR, hazard ratio; ICI, immune checkpoint inhibitor; IMDC, International Metastatic RCC Database Consortium; IO, immuno-oncology; irAE, immune-related adverse event; KIM-1, kidney injury molecule-1; mo, months; M1 NED, M1 with no evidence of disease; NR, not reported; ORR, objective response rate; OS, overall survival; pCR, pathological complete response; PD-1, programmed cell death protein 1; PD-L1, programmed death-ligand 1; PFS, progression-free survival; PPE, palmar-plantar erythrodysesthesia; RCC, renal cell carcinoma; RFS, recurrence-free survival; TFS, treatment-free survival; TKI, tyrosine kinase inhibitor; TRAE, treatment-related adverse event.

### 3.4 Prostate cancer

#### 3.4.1 Localized and biochemically recurrent disease

Prostate cancer has historically been considered an immunologically “cold” tumor, characterized by low somatic mutation rates, limited T-cell infiltration, and a highly immunosuppressive microenvironment dominated by Treg, MDSC, and transforming growth factor-β. However, recent evidence has revealed that ADT can enhance T-cell infiltration into the tumor microenvironment and that radiotherapy can induce immunogenic cell death and promote antigen release, providing a rationale for combining these standard modalities with immunotherapeutic agents. The presence of tissue-restricted antigens such as PAP and PSA offers viable targets for vaccine-based approaches. Moreover, Fc-optimized antibodies targeting CTLA-4 may overcome the limitations of first-generation checkpoint inhibitors by depleting tumor-infiltrating Treg through antibody-dependent cellular cytotoxicity. The CAN-2409 viral immunotherapy trial and the NeoRED-P neoadjuvant anti-CTLA-4 trial now provide proof-of-concept that immunotherapy can modulate the prostate tumor microenvironment in early-stage disease, opening new avenues for curative-intent combinations (1, 2).

##### 3.4.1.1 Viral immunotherapy

The CAN-2409 trial represents a novel immunotherapeutic approach for localized prostate cancer, testing an *in-situ* viral gene therapy combined with standard-of-care radiotherapy. CAN-2409 is a replication-defective adenovirus encoding the herpes simplex virus thymidine kinase (HSV-tk) gene. When injected directly into the prostate and followed by oral valacyclovir (prodrug), it leads to immunogenic cell death of transduced cancer cells, thereby inducing a systemic antitumor immune response. This phase III, multicenter, double-blind, placebo-controlled trial enrolled 745 patients with intermediate- or high-risk localized prostate cancer (by NCCN criteria) who were planning to receive EBRT with or without short-course ADT (≤6 months). Patients were randomized 2:1 to receive three intraprostatic injections of CAN-2409 (5×10¹¹ viral particles/2 mL) or placebo, each followed by 14 days of oral valacyclovir. All patients underwent standard EBRT (with or without ADT) per protocol. With a median follow-up of 50.3 months, the CAN-2409 group significantly reduced the risk of recurrence or death by 30% compared with placebo (median DFS not reached vs. 86.1 months; HR 0.70; 95% CI 0.52–0.94; p=0.0155). Secondary endpoints also favored CAN-2409: the proportion of patients achieving a PSA nadir was 67.1% versus 58.6% (p=0.0164), and pCR rates on 2-year post-treatment biopsies were 80.4% versus 63.6% (p=0.0015). TRAE were mostly grade 1–2 and transient, including chills (33.4% vs. 8.6%), flu-like symptoms (30.5% vs. 13.8%), and fever (25.1% vs. 3.9%). SAEs were uncommon (5.8% vs. 7.3%), and treatment-related SAEs occurred in only 1.7% versus 2.2% of patients. By significantly improving DFS, increasing pCR, and showing a favorable safety profile, the addition of CAN-2409 to standard radiotherapy (with or without short-course ADT) represents the first potentially new therapy for patients with intermediate- and high-risk localized prostate cancer, offering an innovative *in situ* immunotherapy approach (62).

##### 3.4.1.2 PROSTVAC in active surveillance

This phase II RCT evaluated PROSTVAC (a poxviral vaccine encoding PSA with three T-cell co-stimulatory molecules) in patients with low- or intermediate-risk prostate cancer on active surveillance. One hundred fifty-four men were randomized 2:1 to PROSTVAC (priming with vaccinia vector, followed by 6 fowlpox boosts over 140 days) or empty fowlpox vector. The primary outcome was change in CD4 and CD8 T-cell infiltration in biopsy tumor tissue. PROSTVAC did not elicit more favorable T-cell responses than empty fowlpox vector, and there was no difference in Gleason upgrading or PSA change between arms. This was the first study reporting the feasibility and acceptability of an immunotherapy intervention in the active surveillance setting, despite negative results (63).

##### 3.4.1.3 Neoadjuvant Fc-enhanced anti-CTLA-4

The phase I/II NeoRED P trial administered neoadjuvant Fc-enhanced anti-CTLA-4 (BMS-986218) plus ADT vs ADT alone before radical prostatectomy in high-risk localized cancer. Twenty-four men with high-risk localized PCa (Gleason Grade Group ≥3, clinical stage T2-T3b) were randomized 1:1 to ADT (degarelix for 3-4 weeks) with or without BMS-986218 (20 mg IV on days 1 and 15) prior to radical prostatectomy. The primary endpoints were safety and feasibility. The combination was safe and feasible, with no grade ≥3 adverse events related to anti-CTLA-4 (grade 3 asymptomatic lipase elevation in one patient). Mechanistic studies revealed reductions in tumor-infiltrating regulatory T cells (TI-Tregs) correlated with CD16a/FCGR3A expression on tumor macrophages. TI-Treg reduction was associated with improved DFS (p=0.03). Fc-enhanced anti-CTLA-4 also augmented T-cell priming, as shown by increased CD39 + 4-1BB+ CD8 T cells in the tumor and increased proliferating CD4 and CD8 T cells in tumor-draining lymph nodes. This trial provided the first-in-human evidence that Fc-engineered anti-CTLA-4 can deplete TI-Tregs in prostate cancer, which first-generation anti-CTLA-4 antibodies (ipilimumab, tremelimumab) fail to do (64).

##### 3.4.1.4 Oligometastatic disease

The phase II EXTEND RCT evaluated metastasis-directed therapy (MDT) plus ADT in oligometastatic prostate cancer (omPC). Patients with 1-5 metastases amenable to MDT received ADT (continuous or intermittent) and were randomized 1:1 to MDT (SBRT, surgery, or ablation to all sites) or ADT alone. In the continuous ADT basket (n=87), median PFS was 47 months with MDT+ADT versus 22 months with ADT alone (HR 0.50; 95% CI 0.23-1.08; one-sided p=0.036). In the combined analysis of continuous and intermittent ADT baskets (n=174), median PFS was 36 vs. 17 months (HR 0.45; 95% CI 0.30-0.69; p<0.001). MDT+ADT also improved radiographic PFS (HR 0.63) and castration resistance-free survival (HR 0.40). Exploratory immune analyses showed that MDT+ADT induced systemic immune activation, including T-cell receptor (TCR) expansion and contraction, which was associated with improved PFS (HR 0.21; 95% CI 0.06-0.76; p=0.02). TCR modulation was also observed in the independent ORIOLE trial of MDT without ADT. This trial demonstrated that adding MDT to ADT improves multiple clinical endpoints and induces systemic antitumor immunity (65).

##### 3.4.1.5 PROSTVAC in non-metastatic CRPC

This phase II trial compared flutamide with or without PROSTVAC in non-metastatic CRPC (nmCRPC). Patients with rising PSA after definitive therapy, normal testosterone, and no radiographic metastases were randomized to flutamide (250 mg three times daily) or flutamide plus PROSTVAC (vaccinia priming followed by fowlpox boosts). The combination did not improve time to treatment failure (6.9 vs. 4.5 months; p=0.38), though both treatments were well tolerated (66).

#### 3.4.2 Metastatic hormone-sensitive prostate cancer

##### 3.4.2.1 Short-course enzalutamide with/without PROSTVAC

A phase II trial evaluated short-course enzalutamide alone and with PROSTVAC in non-metastatic hormone-sensitive prostate cancer (nmHSPC). Thirty-eight patients with biochemically recurrent PCa, normal testosterone, and no radiographic metastases were randomized 1:1 to enzalutamide (160 mg daily) for 3 months with or without PROSTVAC (6 doses). The primary endpoint was PSA growth kinetics after enzalutamide discontinuation. The addition of PROSTVAC did not improve PSA growth rates, but the impact of short-course enzalutamide alone was remarkable: median PSA decline was >99% (range 84-99%) after 3 months of enzalutamide without ADT, and median time to PSA recovery to baseline was 224 days after treatment cessation. A second course of enzalutamide in 26 patients produced a median PSA decline of >99% again, with median recovery of 189 days. Immune profiling showed that enzalutamide increased NK cells and naïve T cells while decreasing MDSC, and increased T-cell receptor excision circles (TRECs), indicating increased thymic output of naïve T cells. This trial demonstrated that intermittent short-course enzalutamide without ADT can provide durable PSA control with minimal toxicity, representing a potential alternative to intermittent ADT for nmHSPC (67).

#### 3.4.3 Metastatic castration-resistant prostate cancer

Metastatic castration-resistant prostate cancer (mCRPC) represents the terminal phase of prostate cancer progression, where standard androgen receptor pathway inhibitors and taxane chemotherapy provide limited survival benefits. The challenge of immunotherapy in mCRPC stems from the tumor’s low mutational burden, limited neoantigen repertoire, and profoundly immunosuppressive microenvironment. However, subsets of patients with DNA damage repair (DDR) defects, high tumor mutational burden, or microsatellite instability exhibit greater susceptibility to checkpoint inhibition, providing a biomarker-driven rationale for patient selection. Cancer vaccines such as sipuleucel-T aim to overcome immune ignorance by activating antigen-presenting cells and inducing T-cell responses against PSA, though their efficacy is modest in unselected populations. Novel approaches, including bispecific T-cell engagers targeting kallikrein-2 and combination strategies integrating immunotherapy with radiotherapy, are now being evaluated to convert “cold” prostate tumors into “hot” tumors amenable to immune-mediated killing. Despite multiple negative phase III vaccine trials, the emergence of novel immunotherapeutic platforms, including viral gene therapy, Fc-enhanced checkpoint inhibitors, and bispecific antibodies, offers renewed hope for this challenging disease (1, 68).

##### 3.4.3.1 Bispecific T-cell engager

A first-in-human phase I trial of pasritamig, a KLK2xCD3 bispecific T-cell engager, enrolled heavily pre-treated mCRPC patients. At the recommended dose, PSA50 response rate was 42.4%, with a manageable safety profile (low rates of cytokine release syndrome). This opens a new class of immunotherapy for prostate cancer (68).

##### 3.4.3.2 ICI combinations

###### 3.4.3.2.1 Nivolumab plus ipilimumab

The checkMate 650 phase II trial evaluated nivolumab plus ipilimumab in a subset of mCRPC patients. Patients were enrolled in two cohorts: pre-chemotherapy (asymptomatic/minimally symptomatic, n=45) and post-chemotherapy (progressing after docetaxel, n=45). All patients received nivolumab (1 mg/kg) plus ipilimumab (3 mg/kg) every 3 weeks for up to 4 doses, followed by nivolumab monotherapy (480 mg every 4 weeks). In the pre-chemotherapy cohort, ORR was 25% (95% CI 11.5-43.4%), with CRs in 2 patients (6.3%), median rPFS 5.5 months, and median OS 19.0 months. In the post-chemotherapy cohort, ORR was 10% (95% CI 2.1-26.5%), with CRs in 2 patients (6.7%), median rPFS 3.8 months, and median OS 15.2 months. Grade 3-4 TRAEs occurred in 42.2% and 53.3%, with four treatment-related deaths (1.8% in each cohort). Exploratory biomarker analyses suggested that higher TMB and DDR mutations were associated with response. This trial demonstrated that dual ICI has activity in a subset of mCRPC patients but with significant toxicity (69).

###### 3.4.3.2.2 Durvalumab with/without tremelimumab

A phase II RCT evaluated durvalumab (1500 mg every 4 weeks) with or without tremelimumab (75 mg every 4 weeks for 4 doses) in mCRPC patients progressing on abiraterone and/or enzalutamide. The ORR was 19.4% (ITT 17.9%) with the combination, and responses were enriched in tumors that were PD-L1-positive or exhibited DDR defects. This trial supported the rationale for ICI in molecularly selected mCRPC (70).

##### 3.4.3.3 Vaccines

Cancer vaccines aim to educate the patient’s immune system to recognize and eliminate tumour cells by presenting tumour-associated antigens to dendritic cells, thereby priming tumour-specific T-cell responses. In prostate cancer, tissue-restricted antigens such as PAP and PSA provide viable targets. However, the immunosuppressive tumour microenvironment, low mutational burden, and the challenge of inducing durable T-cell responses have limited vaccine efficacy in unselected populations. While sipuleucel-T remains the only FDA-approved cancer vaccine, subsequent phase III trials have largely failed to replicate promising phase II results, highlighting the difficulty of translating vaccine efficacy from early-phase to registrational studies (1).

###### 3.4.3.3.1 Antigen-targeted vaccines: sipuleucel-T

The paradigm for cellular immunotherapy in prostate cancer was established by sipuleucel-T, an autologous product generated by culturing a patient’s peripheral blood mononuclear cells with a recombinant fusion protein of PAP and GM-CSF, which activates antigen-presenting cells to prime T-cell responses. In the landmark IMPACT phase III RCT, 512 patients with asymptomatic or minimally symptomatic mCRPC with Gleason score ≤7 (later amended to any Gleason score) and no visceral metastases were randomized 2:1 to sipuleucel-T (three infusions every 2 weeks) or placebo (control product made from unstimulated cells) (1). Sipuleucel-T reduced the risk of death by 22% (HR 0.78; 95% CI 0.61-0.98; p=0.03), with median OS of 25.8 vs. 21.7 months (4.1-month improvement). The 36-month survival probability was 31.7% vs. 23.0%. No effect on time to disease progression was observed. Adverse events included chills (51.2%), fever (22.5%), and headache (10.7%), mostly grade 1-2. Subgroup analyses showed that patients with lower baseline PSA derived greater benefit (lowest PSA quartile ≤22.1 ng/mL: HR 0.51; 95% CI 0.31-0.85; 13.0-month median OS improvement) (71). Updated analyses showed that control-arm crossover to salvage immunotherapy may have underestimated the true OS benefit, with IPE modeling estimating the sipuleucel-T treatment effect between 3.9 and 8.1 months (72). Long-term immunologic studies demonstrated that sipuleucel-T induces persistent B-cell responses and humoral antigen spread to secondary tumor antigens (PSA, KLK2, K-Ras, LGALS3), with anti-PSA and anti-LGALS3 IgG responses associated with improved OS (anti-PSA: HR 0.38; p=0.01; anti-LGALS3: HR 0.25; p=0.01) (73). Attempts to augment sipuleucel-T with recombinant IL-7 expanded CD4+, CD8+, and NK cells (2.3- to 2.6-fold) and enhanced antigen-specific humoral and T-cell responses. However, the primary IFN-γ ELISpot endpoint was not met, likely due to robust baseline responses from sipuleucel-T alone and the small sample size (n=54). Post-hoc analysis suggested a clinical signal, with more IL-7-treated patients having a PSADT >6 months (31% vs 14%). This first-in-disease study established the feasibility of IL-7 as an immune adjuvant, though optimal dosing and patient selection require further investigation (74).

###### 3.4.3.3.2 Viral vector vaccines: *PROSTVAC*

PROSTVAC employs a heterologous prime-boost strategy using vaccinia and fowlpox vectors encoding PSA with three T-cell co-stimulatory molecules (B7-1, ICAM-1, LFA-3) to enhance antigen presentation and T-cell activation. Despite promising phase II results, the phase III RCT of PROSTVAC in asymptomatic or minimally symptomatic mCRPC failed to meet its primary endpoint. A total of 1257 patients were randomized 1:1:1 to PROSTVAC-V (vaccinia priming) plus PROSTVAC-F (fowlpox boosts), PROSTVAC-F plus GM-CSF, or placebo. Median OS was 34.4 months with PROSTVAC vs. 34.3 months with placebo (HR 1.01; 95% CI 0.83-1.23; p=0.47), despite induction of PSA-specific T-cell responses. This negative trial contrasted with positive phase II results, highlighting the difficulty of translating vaccine efficacy from phase II to phase III (75).

###### 3.4.3.3.3 Personalized peptide vaccination

This approach selects up to four peptides from a warehouse of cancer-associated antigens based on each patient’s HLA type and pre-existing peptide-specific IgG levels, aiming to overcome immune response heterogeneity. A phase II RCT evaluated PPV plus low-dose dexamethasone versus dexamethasone alone in chemotherapy-naive CRPC. Patients with HLA-A2, A24, or A03 positivity and pre-existing peptide-specific IgG responses received up to 4 peptides selected from a warehouse of 24 cancer antigens. The PPV plus dexamethasone arm (n=37) showed improved PSA PFS (22.0 vs. 7.0 months; HR 0.389; p=0.0076) and OS (73.9 vs. 34.9 months; HR 0.412; p=0.00084) compared with dexamethasone alone (n=36). However, the phase III RCT of PPV in HLA-A24-positive patients with CRPC progressing after docetaxel (n=306, randomized 2:1 to PPV or placebo) did not prolong OS (16.1 vs. 16.9 months; HR 1.04; 95% CI 0.80-1.37; p=0.77). Subgroup analysis suggested that patients with lower neutrophil proportion (<64%) or higher lymphocyte proportion (≥26%) at baseline derived benefit (OS HR 0.55 and 0.70, respectively) (76, 77).

###### 3.4.3.3.4 Novel vaccine platforms: GX301, and DCVAC/PCa

Emerging platforms target universal tumour antigens or employ dendritic cell loading. GX301, a telomerase-based vaccine targeting hTERT, was evaluated in a phase II trial with three schedules (8, 4, or 2 administrations) in mCRPC patients stable after docetaxel. The vaccine was safe, with an overall immune responder rate of 54% and 95% of patients showing at least one vaccine-specific response. Immune responses were dose-dependent (65% with 8 doses vs 42% with 2 doses), supporting schedules with more administrations for future trials (78). Conversely, the DCVAC/PCa phase III trial, using autologous dendritic cells loaded with killed LNCaP prostate cancer cells, failed to improve OS when added to docetaxel/prednisone in 1, 182 mCRPC patients (23.9 vs 24.3 months; HR 1.04; p=0.60), adding to the list of negative phase III vaccine trials in mCRPC (79). These contrasting results highlight the ongoing challenges in vaccine development: optimizing antigen selection, overcoming immunosuppression, and identifying patients most likely to benefit.

##### 3.4.3.4 Herbal medicines with PPV

A randomized phase II trial evaluated the addition of herbal medicines (Hochu-ekki-to TJ-41 and Keishi-bukuryo-gan TJ-25) to PPV in CRPC patients. Seventy patients were randomized 1:1 to PPV plus herbal medicines or PPV alone for 8 weeks. While the combination did not improve antigen-specific IgG or CTL responses, PFS, or OS, it stabilized the frequency of monocytic myeloid-derived suppressor cells (Mo-MDSC) and serum IL-6 levels during treatment (PPV+HM: Mo-MDSC 1.91% to 1.92%, p=0.96; IL-6 19.2 to 16.1 pg/mL, p=0.63; PPV alone: Mo-MDSC 0.91% to 1.49%, p=0.012; IL-6 9.2 to 19.4 pg/mL, p=0.04). Patients without an increase in Mo-MDSC had significantly longer survival (32.6 vs. 12.9 months; HR 0.48; p=0.026), suggesting that herbal medicines may prevent immunotherapy-induced immunosuppression (80).

The full list of prostate cancer trials, with their key efficacy and safety results, is presented in **Table 3**.

**Table:** Table 3 Evolving immunotherapy strategies in prostate cancer: vaccines, checkpoints, and emerging combinations.

Trial Name/reference	Phase	Population	Experimental arm	Control arm	Primary endpoint(s)	Key efficacy results	Key safety/tolerability results
CAN-2409 (62)	III	Intermediate/high-risk localized PrCa	CAN-2409 + valacyclovir + EBRT ± short-course ADT	Placebo + EBRT ± ADT	DFS	DFS HR 0.70; p=0.0155; pCR 80.4% vs 63.6%	Grade 1-2: chills 33.4%, fever 25.1%; SAEs 5.8% vs 7.3%
PROSTVAC (AS) (63)	II	Low/intermediate-risk PrCa on AS	PROSTVAC	Empty vector	Change in CD4/CD8 infiltration	No difference; first immunotherapy feasibility in AS	Well tolerated
Neoadjuvant anti-CTLA-4 + ADT (64)	I	High-risk localized PrCa	BMS-986218 (Fc-enhanced anti-CTLA-4) + ADT	ADT alone	Safety/Treg reduction	Feasible; reduced TI-Tregs; associated with improved DFS	No grade ≥3 AEs related to anti-CTLA-4
EXTEND (65)	II	Oligometastatic PrCa	MDT + ADT	ADT alone	PFS	PFS HR 0.45; median PFS 36 vs 17 mo; TCR expansion observed	Not reported
PROSTVAC (nmCRPC) (66)	II	Non-metastatic CRPC	Flutamide + PROSTVAC	Flutamide alone	Time to treatment failure	No improvement (6.9 vs 4.5 mo)	Well tolerated
Enzalutamide + PROSTVAC (67)	II	Non-metastatic CSPC	Enzalutamide (3 mo) + PROSTVAC	Enzalutamide alone	PSA kinetics	PSA decline >99%; immune changes (NK ↑, MDSC ↓)	Minimal toxicity
Pasritamig (68)	I	Heavily pre-treated mCRPC	Pasritamig (KLK2xCD3 bispecific)	—	Safety/PSA50	PSA50 42.4%; manageable CRS	Manageable CRS
CheckMate 650 (69)	II	mCRPC (pre-chemo and post-docetaxel)	Nivolumab + ipilimumab	—	ORR	Pre-chemo: ORR 25%, rPFS 5.5 mo, OS 19.0 mo; Post-chemo: ORR 10%	Grade 3-4 TRAEs 42-53%; 4 treatment-related deaths
Durvalumab ± tremelimumab (70)	II	mCRPC post-abiraterone/enzalutamide	Durvalumab + tremelimumab	Durvalumab alone	ORR	ORR 19.4% (ITT 17.9%); enriched in PD-L1+ or DDR-mutant	Not reported
IMPACT (sipuleucel-T) (1, 71–74)	III	Asymptomatic/minimally symptomatic mCRPC	Sipuleucel-T	Placebo	OS	OS HR 0.78; median OS 25.8 vs 21.7 mo; 36-mo OS 31.7% vs 23.0%	Grade 1-2: chills 51.2%, fever 22.5%; no progression effect
PROSTVAC Phase III (75)	III	Asymptomatic/minimally symptomatic mCRPC	PROSTVAC (± GM-CSF)	Placebo	OS	OS HR 1.01; p=0.47 (negative)	Not reported
PPV + low-dose dexamethasone (76)	II	Chemotherapy-naïve CRPC	PPV + dexamethasone	Dexamethasone alone	PSA PFS	PSA PFS HR 0.389; OS HR 0.412	Not reported
PPV Phase III (77)	III	CRPC post-docetaxel	PPV	Placebo	OS	OS HR 1.04; p=0.77 (negative)	Not reported
GX301 (78)	II	mCRPC post-docetaxel	Telomerase vaccine (GX301) – 3 schedules	Comparison of 8 vs 4 vs 2 doses	Immunological response	54% responder rate; dose-dependent (65% with 8 doses vs 42% with 2)	No major side effects
DCVAC/PCa (79)	III	mCRPC	DCVAC/PCa + docetaxel/prednisone	Placebo + docetaxel/prednisone	OS	OS HR 1.04; p=0.60 (negative)	Not reported
IL-7 + sipuleucel-T (81)	II	mCRPC post-sipuleucel-T	rhIL-7 (CYT107)	Observation	Immune response (ELISpot)	Lymphocyte expansion 2.3-2.6×; primary endpoint not met	Not reported
Herbal medicines + PPV (80)	II	CRPC	PPV + Hochu-ekki-to/Keishi-bukuryo-gan	PPV alone	Immunological efficacy	Stabilised MDSC and IL-6; no survival benefit	Not reported
CA184-043 (82)	III	Post-docetaxel mCRPC with bone metastases	Ipilimumab + single-fraction RT	Placebo + RT	OS	5-yr OS 7.9% vs 2.7%; 3-yr 15.3% vs 7.9%	G3-4 immune-related early; crossing survival curves

ADT, androgen deprivation therapy; AS, active surveillance; CR, complete response; CRPC, castration-resistant prostate cancer; CRS, cytokine release syndrome; CSPC, castration-sensitive prostate cancer; DDR, DNA damage repair; DFS, disease-free survival; EBRT, external beam radiotherapy; GM-CSF, granulocyte-macrophage colony-stimulating factor; HR, hazard ratio; IL-7, interleukin-7; MDSC, myeloid-derived suppressor cell; MDT, metastasis-directed therapy; mCRPC, metastatic castration-resistant prostate cancer; mo, months; NK, natural killer; nmCRPC, non-metastatic castration-resistant prostate cancer; NR, not reported; ORR, objective response rate; OS, overall survival; pCR, pathological complete response; PD-1, programmed cell death protein 1; PD-L1, programmed death-ligand 1; PFS, progression-free survival; PPV, personalized peptide vaccination; PrCa, prostate cancer; PSA, prostate-specific antigen; rhIL-7, recombinant human interleukin-7; rPFS, radiographic progression-free survival; RT, radiotherapy; SAE, serious adverse event; TCR, T-cell receptor; TRAE, treatment-related adverse event; Treg, regulatory T cell.

### 3.5 Immunotherapy combined with radiotherapy in urological cancers

The synergistic potential of radiotherapy and immunotherapy has emerged as a promising treatment paradigm across urological malignancies. Radiation-induced immunogenic cell death, neoantigen release, and modulation of the tumour microenvironment can transform “cold” tumours into “hot” ones amenable to checkpoint inhibition. However, optimal fractionation, sequencing, and patient selection remain critical determinants of success (83, 84).

#### 3.5.1 Bladder cancer

In MIBC, bladder-preserving strategies have made significant progress with immunoradiotherapy combinations. The phase II IMMUNOPRESERVE trial evaluated durvalumab plus tremelimumab with concurrent radiotherapy in patients with non-metastatic MIBC (nmMIBC). The regimen achieved a 81% CR rate, with 2-year bladder-intact DFS of 65% and manageable grade 3–4 toxicity in 31% of patients (85). Similarly, the INDIBLADE study employed induction ipilimumab/nivolumab followed by chemoradiotherapy, reporting 2-year bladder preservation event-free and OS rates of 78% and 96%, respectively. Importantly, clearance of ctDNA post-induction immunotherapy was identified as a predictive biomarker for favourable outcomes (86).

The phase II PCR-MIB (ANZUP 1502) trial evaluated pembrolizumab with concomitant cisplatin-based chemoradiation, achieving a CR rate of 88% at 24 weeks and 2-year distant metastasis-free survival of 78%. However, predefined unacceptable toxicity occurred in 32% of patients, highlighting the need for careful patient selection (87). These findings are currently being validated in the phase III KEYNOTE-992 trial, comparing pembrolizumab plus chemoradiation versus chemoradiation alone (88).

Important safety considerations emerged from the PLUMMB trial, which was prematurely terminated due to dose-limiting urinary toxicities from hypofractionated radiotherapy (36 Gy in 6 fractions) combined with pembrolizumab, suggesting that hypofractionated regimens should be avoided in this setting (89).

In BCG-unresponsive NMIBC, the ADAPT-BLADDER study demonstrated that durvalumab with external beam radiotherapy (6 Gy × 3) was feasible, with CR rates of 50% and 33% at 3 and 12 months, respectively. However, this combination was associated with higher grade 3–4 toxicity (42%) compared to durvalumab plus BCG therapy (15%), tempering enthusiasm for this approach (90).

For cisplatin-ineligible MIBC patients, several phase II trials have explored immunotherapy with bladder-sparing radiotherapy. Mouw et al. reported that avelumab with bladder radiotherapy (55 Gy in 20 fractions or 65 Gy in 35 fractions with nodal irradiation) achieved clinical CR in 64% of patients, with a median OS of 30.9 months and only 21% grade 3 toxicity, notably in an elderly population with a median age of 83 years and high comorbidity scores (91). These results compare favourably with the DUART trial (durvalumab with bladder and nodal RT, clinical CR 62%, median PFS 22 months) (92) and the NUTRA trial (nivolumab with concurrent and adjuvant RT, median PFS 18 months) (93).

In mUC, the phase I/II trial by Sundahl et al. randomized patients to pembrolizumab with stereotactic body radiotherapy (SBRT; 24 Gy in three fractions) administered either before the first or third pembrolizumab cycle. The concomitant regimen produced a markedly higher ORR (44.4%) compared to the sequential arm (0%), indicating that the timing of radiation administration is critical for stimulating systemic immune responses. Treatment effect correlated with ctDNA fraction reduction, offering a potential surrogate marker for treatment efficacy (94).

#### 3.5.2 Prostate cancer

In prostate cancer, the ICE-PAC trial evaluated avelumab combined with stereotactic ablative body radiotherapy (SABR; 20 Gy single fraction) in mCRPC, achieving a disease control rate of 48% and ORR of 31%, with abscopal effects observed in both irradiated and non-irradiated lesions (95). This builds on earlier phase I/II data where ipilimumab 10 mg/kg with radiotherapy (8 Gy single fraction to bone lesions) achieved at least a 50% decline in PSA levels in 16% of patients, with a single complete response (96).

The phase III CA184–043 trial ultimately demonstrated that ipilimumab after bone radiotherapy (8 Gy) led to prolonged survival in post-docetaxel mCRPC. Despite early intersection of survival curves due to immune-related toxicities, the 3-year, 4-year, and 5-year OS rates were two to three times higher with ipilimumab compared to placebo plus radiotherapy (15.3% vs 7.9%, 10.1% vs 3.3%, and 7.9% vs 2.7%, respectively), identifying a subset of patients deriving durable benefit (97).

In localized or oligometastatic grade group 5 prostate cancer, Bryant et al. assessed nivolumab with high-dose-rate brachytherapy (two implants of 11.5 Gy), ADT, and external beam radiotherapy (45 Gy in 25 fractions). Freedom from biochemical recurrence at 2 years was 90.3%, exceeding the prespecified historical control value of 75% (p=0.024). The Decipher immunosuppression score (RIS) proved effective as a biomarker, with higher RIS associated with early pathological response (p=0.005) and independently predictive of time to metastatic failure (98). This represents an improvement over the earlier phase I proof-of-concept trial by Yuan et al., which demonstrated safety and feasibility, with 3 of 6 patients showing early pathological responses and evidence of immune infiltration (CD8+ T cells, CD4+ effector T cells) (99).

Conversely, the randomized phase II trial by Twardowski et al. comparing sipuleucel-T with or without prior radiation sensitization (300 cGy/day up to 3000 cGy total) showed no effect of irradiation on humoral or cellular immune responses. While PFS demonstrated a nonsignificant trend favouring the irradiated arm (3.65 vs 2.46 months; p=0.06), the cumulative upregulation of CD54+ cells was lower in the irradiated arm, suggesting a short-lived suppressive effect of radiation on antigen-presenting cell activation (100).

#### 3.5.3 Kidney cancer

In oligometastatic clear cell RCC, the RAPPORT study assessed SABR (single fraction 20 Gy or 30 Gy in 10 Gy fractions) with short-course pembrolizumab (200 mg every 3 weeks for 8 cycles, lasting 6 months). Among 30 evaluable patients with a median age of 62 years, the 2-year freedom from local recurrence was 92%, ORR 63%, disease control rate 83%, and 2-year overall survival 74%. Grade 3 treatment-related toxicity occurred in 13% (pneumonitis, dyspnoea, liver enzyme elevation), with no grade 4–5 events, and 80% of patients received all cycles of immunotherapy. Median PFS was 15.6 months (101). These outcomes compare favourably with the NIVES study (single-site irradiation with nivolumab in disseminated metastatic disease: ORR 17%, DCR 55%, median PFS 5.6 months), suggesting that ablating all metastases may be more effective than irradiating one site only in oligometastatic patients (102).

In a phase II study by Bulgarelli et al., high-dose interleukin-2 (18 MIU/m²/day for 72 hours continuous infusion, up to 6 cycles) was evaluated after irradiation of 1–5 non-index metastases (3 fractions of 6–12 Gy). Among 19 patients (9 RCC, 10 melanoma), 52.6% had tumour control with 3 partial responses and seven stable diseases at a median follow-up of 39.2 months. Immunological responses by IFN-γ ELISPOT were seen in 84.2% of patients, with increases in circulating CD4+ effector memory T cells. The regimen had minimal side effects with no ICU admissions required, supporting the feasibility of shorter IL-2 schedules with corticosteroid prophylaxis (103).

Ongoing phase I trials are evaluating triplet therapy comprising atezolizumab, tiragolumab (anti-TIGIT), and SBRT (3 × 8 Gy) in metastatic bladder cancer, RCC, non-small cell lung cancer, and head and neck cancers, suggesting that multi-agent immunoradiotherapy combinations are being actively explored (104).

Overall, these trials demonstrate that immunoradiotherapy regimens are feasible across urological cancers, but optimal fractionation schedules, sequencing, and patient selection criteria remain areas of active investigation. The importance of avoiding hypofractionated regimens in bladder cancer, leveraging ctDNA as a predictive biomarker (INDIBLADE, Sundahl), and the potential of transcriptomic signatures are emerging themes that will inform future trial design.

The key immunoradiotherapy trials are summarized in **Table 4**.

**Table:** Table 4 Key trials of immunotherapy combined with radiotherapy in urological cancers.

Trial name/reference	Phase	Population (subtype, stage)	Experimental arm	Control arm	Primary endpoint(s)	Key efficacy results	Key safety/tolerability results
IMMUNOPRESERVE (85)	II	MIBC	Durvalumab + tremelimumab + concurrent RT	—	CR	CR 81%; 2-yr BIDFS 65%	Grade 3-4 toxicity 31%
INDIBLADE (86)	II	MIBC	Ipilimumab + nivolumab (induction) + chemo-RT	—	BEFS	2-yr BEFS 78%; OS 96%; ctDNA clearance predictive	Not reported
PCR-MIB (ANZUP 1502) (87)	II	MIBC	Pembrolizumab + concurrent chemo-RT	—	CR	CR 88% at 24 wks; 2-yr DMFS 78%	32% unacceptable toxicity
PLUMMB (89)	I/II	MIBC	Pembrolizumab + hypofractionated RT (36 Gy/6 fx)	—	Safety	Terminated early	Dose-limiting urinary toxicity
ADAPT-BLADDER (90)	I	BCG-unresponsive NMIBC	Durvalumab + EBRT (6 Gy × 3)	Durvalumab + BCG	Safety/CR	CR 50% at 3 mo; 33% at 12 mo	Grade 3-4 42% vs 15% (BCG arm)
Mouw et al. (91)	II	Cisplatin-ineligible MIBC	Avelumab + bladder RT (55 Gy/20 fx or 65 Gy/35 fx)	—	CR	CR 64%; median OS 30.9 mo	Grade 3 toxicity 21%; no grade 4-5
DUART (92)	II	MIBC	Durvalumab + bladder + nodal RT	—	CR	CR 62%; median PFS 22 mo	Not reported
NUTRA (93)	II	Node-negative MIBC	Nivolumab + concurrent + adjuvant RT	—	PFS	Median PFS 18 mo	Not reported
Sundahl et al. (94)	I/II	Metastatic UC	Pembrolizumab + concomitant SBRT	Pembrolizumab + sequential SBRT	Safety/ORR	ORR 44.4% (concurrent) vs 0% (sequential); ctDNA correlates	G3 lymphopenia (1 pt)
ICE-PAC (95)	II	mCRPC	Avelumab + SABR (20 Gy/1 fx)	—	DCR	DCR 48%; ORR 31%; abscopal effects	Manageable
Phase I/II ipilimumab (96)	I/II	mCRPC	Ipilimumab 10 mg/kg + bone RT (8 Gy)	—	PSA response	PSA decline ≥50% in 16%; 1 CR	Manageable
CA184-043 (97)	III	Post-docetaxel mCRPC with bone metastases	Ipilimumab + bone RT (8 Gy)	Placebo + RT	OS	5-yr OS 7.9% vs 2.7%; 3-yr 15.3% vs 7.9%	G3-4 immune-related early; crossing survival curves
Bryant et al. (98)	II	Localized/oligometastatic GG5 PrCa	Nivolumab + HDRBT (2×11.5 Gy) + EBRT (45 Gy) + ADT	—	FFBR	2-yr FFBR 90.3% (exceeded 75% historical control; p=0.024); RIS biomarker predictive	G2 6.3%; G3 6.3%; G4 + 0%
Yuan et al. (99)	I	GG5 PrCa	Nivolumab + HDRBT + EBRT + ADT	—	Safety	Early response 3/6; immune infiltration ↑ (CD8+, CD4+ effector)	1 G3 autoimmune hepatitis
Twardowski et al. (100)	II	mCRPC	Sipuleucel-T + prior RT (300 cGy/day to 3000 cGy)	Sipuleucel-T alone	Immune response	PFS 3.65 vs 2.46 mo (p=0.06, trend); no effect on immune responses	Well tolerated
RAPPORT (101)	II	Oligometastatic ccRCC	Pembrolizumab (short-course) + SABR (20 Gy/1 fx or 30 Gy/10 fx)	—	FFLP	2-yr FFLP 92%; ORR 63%; DCR 83%; OS 74%; PFS 15.6 mo	Grade 3 13% (pneumonitis, dyspnoea, ALP/ALT); no grade 4-5
NIVES (102)	II	Pre-treated mRCC	Nivolumab + SBRT (single site)	—	ORR	ORR 17%; DCR 55%; PFS 5.6 mo	Rare
Bulgarelli et al. (103)	II	RCC + Melanoma	HD IL-2 + RT (3×6-12 Gy, non-index fields)	—	DCR	DCR 52.6%; PR 15.7%; immune response 84.2%	Frequent but reversible; no ICU
Phase I multi-cohort (104)	I	Metastatic bladder, NSCLC, RCC, HNSCC	Atezolizumab + tiragolumab + SBRT (3×8 Gy)	—	Safety	Ongoing	Ongoing

AE, adverse event; BEFS, bladder preservation event-free survival; BIDFS, bladder-intact disease-free survival; CR, complete response; CRS, cytokine release syndrome; DCR, disease control rate; DFS, disease-free survival; DLT, dose-limiting toxicity; DMFS, distant metastasis-free survival; EBRT, external beam radiotherapy; EFS, event-free survival; FFBR, freedom from biochemical recurrence; FFLP, freedom from local progression; GC, gemcitabine-cisplatin; GG5, Grade Group 5; HDRBT, high-dose-rate brachytherapy; IO, immuno-oncology; MIBC, muscle-invasive bladder cancer; mo, months; MPR, major pathologic response; NED, no evidence of disease; NMIBC, non-muscle-invasive bladder cancer; NR, not reported; ORR, objective response rate; OS, overall survival; pCR, pathological complete response; PD-L1, programmed death-ligand 1; PFS, progression-free survival; PPE, palmar-plantar erythrodysesthesia; PrCa, prostate cancer; QoL, quality of life; RCC, renal cell carcinoma; rPFS, radiographic progression-free survival; RT, radiotherapy; SABR, stereotactic ablative body radiotherapy; SBRT, stereotactic body radiotherapy; TFS, treatment-free survival; TRAE, treatment-related adverse event; UC, urothelial carcinoma; wks, weeks; yr, year.

### 3.6 Real-world evidence in immunotherapy for urological cancers

While randomized controlled trials establish foundational efficacy under idealized conditions, real-world data (RWD) provide critical insights into treatment effectiveness, safety, and patient outcomes in diverse, unselected clinical populations. The integration of RWD has become increasingly important to bridge the efficacy-effectiveness gap, particularly as immunotherapy paradigms evolve across kidney, bladder, and prostate cancers.

#### 3.6.1 Advanced and metastatic urothelial carcinoma

Multiple large-scale real-world studies have evaluated ICI utilization and outcomes in la/mUC. The ARON-2 global study demonstrated that real-world effectiveness of pembrolizumab after platinum-based chemotherapy approximates trial data, with median OS of 10.2 months, though with considerable heterogeneity in patient characteristics and subsequent therapies. Notably, the addition of SBRT to pembrolizumab was associated with improved 1-year OS rates (61% vs. 47% without radiotherapy), suggesting potential synergistic effects in selected patients (105). However, a Dutch nationwide cohort comparison with KEYNOTE-045 revealed significant disparities: the real-world population comprised more patients with ECOG performance status ≥2 (10.3% vs. 1.1%), hemoglobin <10 g/dL (29.8% vs. 16.4%), and carboplatin-based chemotherapy exposure (49.0% vs. 25.9%), resulting in substantially shorter median OS (5.5 vs. 10.1 months) despite comparable objective response rates (106). These findings underscore the importance of patient selection and the prognostic impact of baseline characteristics in routine practice.

The introduction of maintenance avelumab following platinum-based chemotherapy has shifted treatment paradigms, though its comparative effectiveness against the previous standard of pembrolizumab at progression remains debated. A Dutch nationwide real-world comparison demonstrated no significant survival improvement following this transition: the adjusted restricted mean survival time (RMST) difference up to 24 months was only 0.7 months (95% CI −1.1 to 2.3), despite higher ICI utilization in the avelumab era (72.3% vs. 56.2%). This suggests that a treatment-free interval after chemotherapy, followed by ICI at progression, may be as effective as immediate maintenance therapy while minimizing toxicity and treatment burden (107). The AVENANCE study, the largest real-world study of avelumab first-line maintenance reported to date, enrolled 595 patients across 82 centers in France and confirmed real-world effectiveness with median OS of 21.3 months from avelumab initiation and 26.5 months from the start of first-line platinum-based chemotherapy. In exploratory analyses, patients receiving second-line enfortumab vedotin after avelumab maintenance achieved median OS of 41.5 months, supporting sequential treatment strategies (108).

Histologic heterogeneity further complicates real-world outcomes. The ARON-2EV study evaluated 222 patients with urothelial carcinoma with squamous differentiation (UCSD) across three treatment cohorts (avelumab, pembrolizumab, enfortumab vedotin), demonstrating consistently poorer outcomes compared to pure urothelial carcinoma: median OS was 13.0 vs. 26.8 months with avelumab, 10.2 vs. 18.5 months with pembrolizumab, and 7.6 vs. 13.1 months with enfortumab vedotin, establishing UCSD as an independent adverse prognostic factor across modern systemic therapies (109).

First-line immunotherapy in cisplatin-ineligible patients has also been characterized in real-world settings. The ARON-2 study of 162 patients receiving frontline pembrolizumab reported median OS of 15.8 months and ORR of 36%, with notable gender disparities (male 21.2 vs. female 11.7 months, p=0.031) and strong prognostic influence of Bajorin risk factors and bone metastases. Performance status emerges as a critical determinant of ICI outcomes across real-world mUC populations, with ECOG PS ≥2 consistently associated with substantially shorter survival regardless of treatment line or setting (110). A Danish nationwide study of 139 patients receiving pembrolizumab in first- and second-line settings reported comparable outcomes to clinical trials (first-line ORR 30.2%, median OS 9.2 months; second-line ORR 27.3%, median OS 9.1 months), without any new toxicities registered (111).

Finally, ICI rechallenge strategies have been evaluated in real-world la/mUC using the TriNetX global database. Among 292 patients receiving sequential ICI therapies, rechallenge ≥12 months after prior ICI in the metastatic setting was associated with longer median OS (34.6 vs. 11.7 months, HR 0.46, p=0.027), while prior ICI exposure in nonmetastatic settings predicted favorable outcomes upon subsequent rechallenge in advanced disease (112). These findings suggest that treatment-free intervals and prior disease stage may inform rechallenge decisions, though prospective trials are essential to validate these hypotheses.

#### 3.6.2 Metastatic renal cell carcinoma

In mRCC, real-world evidence has illuminated optimal integration of immunotherapy with local therapies. The Canadian Kidney Cancer Information System (CKCis) database evaluated the timing of cytoreductive nephrectomy (CN) relative to immunotherapy-based systemic treatment (ST) in 588 patients. Patients receiving ST followed by deferred CN (ST-CN) demonstrated superior OS (HR 0.30) and PFS (HR 0.45) compared to ST-only, with higher ORR (59.6% vs. 32%) and disease control rates (71.9% vs. 52.8%). These findings suggest that patient selection for deferred CN after initial systemic therapy response may optimize outcomes in the contemporary immunotherapy era, though residual confounding and selection bias require cautious interpretation (113).

The specific value of immunotherapy in non-clear cell histologies was further explored in metastatic papillary RCC, where IO-based regimens demonstrated significantly longer time-to-treatment failure (9.9 vs. 5.9 months, HR 0.62) and OS (36.9 vs. 23.2 months, HR 0.54) compared to TKI monotherapy, with the TKI-IO combination showing the most favorable outcomes (TTF 16.9 months, OS not reached) (114). Spanish real-world evidence from the SOGUG cohort of 222 patients confirmed nivolumab monotherapy efficacy in previously treated advanced RCC, with median OS of 18.1 months, PFS of 4.96 months, and ORR of 23%, consistent with CheckMate-025 trial data; poor IMDC risk score and ≥3 metastatic sites independently predicted worse outcomes, while prior nephrectomy was associated with improved survival (115).

The RAPPORT trial, a prospective multi-institutional phase I/II study, evaluated stereotactic ablative body radiotherapy (SABR) followed by short-course pembrolizumab in 30 patients with oligometastatic clear cell RCC. The combination was well tolerated with 13% grade 3 treatment-related adverse events and no grade 4–5 events. Two-year freedom from local progression was 92%, ORR 63%, disease control rate 83%, and 2-year OS 74%, with median PFS of 15.6 months. These results suggest that total metastatic ablation with radiotherapy combined with abbreviated immunotherapy may provide durable disease control in selected patients with low-volume metastatic disease (116).

#### 3.6.3 Prostate cancer

In prostate cancer, real-world evidence for immunotherapy remains limited given the rarity of microsatellite instability-high (MSI-H) disease. The Sipuleucel-T has demonstrated significant survival benefits in combination with androgen receptor-targeting agents (ARTAs). A Medicare-based analysis of 773 men receiving Sipuleucel-T plus ARTA versus 4, 642 receiving ARTA monotherapy showed median OS of 30.4 versus 14.3 months, representing a 28.3% reduction in mortality risk (HR 0.717), supporting the strategic value of combining immunotherapy with hormonal agents through distinct mechanisms of action (117). The PROCEED registry, a prospective observational study of 1, 976 patients, confirmed contemporary real-world survival data with median OS of 30.7 months, with 77.1% receiving subsequent anticancer interventions and 32.5% experiencing 1-year treatment-free intervals after Sipuleucel-T (118).

#### 3.6.4 Perioperative and bladder-sparing strategies

The perioperative treatment landscape has evolved with adjuvant immunotherapy integration. A Japanese real-world study of 1165 patients with MIBC and 4401 with upper tract urothelial carcinoma (UTUC) undergoing radical surgery found that neoadjuvant chemotherapy was common in MIBC (57.6%) but uncommon in UTUC (5.5%), while adjuvant therapy was less frequent overall. Following nivolumab approval, adjuvant immunotherapy rapidly became the predominant component in MIBC (75.4% of adjuvant treatments), whereas uptake remained limited in UTUC (0.9%). Renal function significantly influenced regimen selection, with higher eGFR associated with cisplatin-based chemotherapy and lower eGFR with carboplatin or nivolumab (119).

Bladder-sparing approaches combining immunotherapy with radiotherapy have shown promising real-world results. A retrospective Chinese study of 25 patients with MIBC unfit for radical cystectomy who received PD-1 inhibitors (tislelizumab or toripalimab) with radiotherapy or chemoradiotherapy achieved clinical complete response in 92%, with 1-year DFS and OS rates of 92% and 96%, respectively. Treatment was feasible and safe, with grade 3–4 adverse events in 35.7% and no treatment discontinuations due to toxicity (120).

For oligometastatic disease, a Swedish single-institution real-world experience of 39 patients with mUC treated with SBRT reported local control rate of 82%, median PFS of 4.1 months, and median OS of 26.2 months. Notably, 15% of patients achieved sustained long-term survival benefit and never required systemic treatments after SBRT, with single metastatic lesions associated with significantly better outcomes than multiple lesions (HR 4.12) (121).

#### 3.6.5 Safety and tolerability in real-world populations

Real-world safety data confirm the favorable tolerability profile of ICIs across diverse populations. An Indian prospective observational study characterized toxicity patterns across ICIs in 53 patients with various malignancies including urothelial bladder cancer, reporting fatigue (73.6%), anemia (62.3%), and pneumonitis (17%) as the most common adverse events, with remarkably low grade 3–4 toxicity (3.77%) and no treatment discontinuations due to toxicity (122). The AVENANCE study reported avelumab-related treatment-emergent adverse events in 55% of patients, with asthenia/fatigue, skin toxicities, gastrointestinal disorders, and endocrine disorders as the most common, confirming no new safety concerns in real-world practice (108).

#### 3.6.6 Treatment patterns and access

Real-world studies reveal critical gaps in treatment access and sequencing. An Austrian tertiary center study of 131 patients with advanced/metastatic UC found that 61% received second-line treatment (double the historical rate), with 75% of these receiving ICIs. Patients staying on immunotherapy for >6 months had median OS of 59 months compared to 14 months for those with <6 months of therapy (HR 0.12). C-reactive protein kinetics and metastatic site location emerged as practical prognostic biomarkers in this setting (123).

Collectively, real-world evidence in urological cancers demonstrates both the translational potential and limitations of immunotherapy beyond clinical trials. While RWD confirms substantial efficacy in selected populations, it also reveals critical gaps in access, the prognostic dominance of baseline performance status and histologic subtype, the potential for radiotherapy-immunotherapy synergy, and the need for refined biomarkers to optimize patient selection across diverse healthcare settings.

The key real-world evidence studies are summarized in **Table 5**.

**Table:** Table 5 Key real-world evidence studies in immunotherapy for urological cancers.

Study/reference	Population	Setting	Immunotherapy	Key findings	Notable insights
ARON-2 (105)	mUC (post-chemo)	Global retrospective	Pembrolizumab	Median OS 10.2 mo; 1-yr OS 61% with SBRT vs 47% without	SBRT associated with improved OS; real-world efficacy approximates trials
Dutch nationwide cohort (106)	mUC	Nationwide retrospective	Pembrolizumab	Median OS 5.5 mo (real-world) vs 10.1 mo (KEYNOTE-045)	Worse PS, anaemia, carboplatin exposure drive poorer outcomes
Dutch maintenance comparison (107)	mUC	Nationwide retrospective	Avelumab maintenance vs pembrolizumab at progression	RMST difference 0.7 mo at 24 mo; no significant OS benefit	Treatment-free interval may be as effective as immediate maintenance
AVENANCE (108)	mUC	French prospective (n=595)	Avelumab maintenance	Median OS 21.3 mo (from avelumab); 26.5 mo (from chemo start)	Second-line EV after avelumab: median OS 41.5 mo
ARON-2EV (109)	UCSD vs pure UC	Global retrospective	Avelumab, pembrolizumab, EV	OS: UCSD vs pure UC: 13.0 vs 26.8 mo (avelumab), 10.2 vs 18.5 mo (pembrolizumab), 7.6 vs 13.1 mo (EV)	UCSD is independent adverse prognostic factor
ARON-2 frontline (110)	Cisplatin-ineligible mUC	Global retrospective	First-line pembrolizumab	Median OS 15.8 mo; ORR 36%; male 21.2 vs female 11.7 mo	Gender disparities; Bajorin risk factors prognostic
Danish nationwide (111)	mUC	Nationwide retrospective	Pembrolizumab (1st/2nd line)	1st line: ORR 30.2%, OS 9.2 mo; 2nd line: ORR 27.3%, OS 9.1 mo	Outcomes comparable to trials; no new toxicities
TriNetX rechallenge (112)	mUC	Global database (n=292)	ICI rechallenge	Rechallenge ≥12 mo: OS 34.6 vs 11.7 mo (HR 0.46)	Longer treatment-free interval predicts better rechallenge outcomes
CKCis (113)	mRCC	Canadian registry (n=588)	IO-based ST ± deferred CN	ST-CN vs ST-only: OS HR 0.30; PFS HR 0.45; ORR 59.6% vs 32%	Deferred CN after IO response may improve outcomes
Papillary RCC (114)	Metastatic papillary RCC	Global retrospective	IO vs TKI vs IO+TKI	IO vs TKI: TTF HR 0.62; OS HR 0.54; IO+TKI best outcomes	IO-based regimens superior in non-clear cell histology
SOGUG cohort (115)	Advanced RCC	Spanish retrospective (n=222)	Nivolumab monotherapy	Median OS 18.1 mo; PFS 4.96 mo; ORR 23%	Consistent with CheckMate-025; IMDC risk and ≥3 sites predictive
RAPPORT (116)	Oligometastatic ccRCC	Prospective phase I/II (n=30)	SABR + short-course pembrolizumab	2-yr FFLP 92%; ORR 63%; DCR 83%; OS 74%; PFS 15.6 mo	Total metastatic ablation + abbreviated IO provides durable control
Medicare analysis (117)	mCRPC	US Medicare database	Sipuleucel-T + ARTA vs ARTA alone	OS 30.4 vs 14.3 mo (HR 0.717); 28.3% mortality reduction	Combining immunotherapy with ARTA improves outcomes
PROCEED registry (118)	mCRPC	Prospective US registry (n=1, 976)	Sipuleucel-T	Median OS 30.7 mo; 77.1% subsequent therapy; 32.5% 1-yr treatment-free	Real-world survival confirms trial data
Japanese perioperative (119)	MIBC/UTUC	Japanese retrospective (n=5, 566)	Adjuvant IO	MIBC: 75.4% adjuvant IO uptake; UTUC: 0.9% uptake	Stark disparities in IO adoption between tumour subtypes
Chinese bladder-sparing (120)	MIBC (unfit for cystectomy)	Chinese retrospective (n=25)	PD-1 inhibitors + RT	CR 92%; 1-yr DFS 92%; OS 96%	Bladder-sparing IO-RT feasible and effective
Swedish SBRT (121)	Oligometastatic mUC	Swedish single-institution (n=39)	SBRT	Local control 82%; PFS 4.1 mo; OS 26.2 mo; 15% never needed systemic therapy	Single lesion predicts better outcomes (HR 4.12)
Indian safety (122)	Various malignancies	Indian prospective (n=53)	ICIs	G3-4 toxicity 3.77%; no treatment discontinuations	Low toxicity in real-world Indian population
Austrian treatment patterns (123)	Advanced UC	Austrian single-center (n=131)	ICIs	61% second-line; 75% received ICI; >6 mo IO: OS 59 vs 14 mo (HR 0.12)	CRP kinetics and metastatic site prognostic

ARTA, androgen receptor-targeting agent; CN, cytoreductive nephrectomy; CR, complete response; CRP, C-reactive protein; DCR, disease control rate; DFS, disease-free survival; EV, enfortumab vedotin; FFLP, freedom from local progression; IO, immuno-oncology; ICI, immune checkpoint inhibitor; MIBC, muscle-invasive bladder cancer; mo, months; mRCC, metastatic renal cell carcinoma; mUC, metastatic urothelial carcinoma; ORR, objective response rate; OS, overall survival; PFS, progression-free survival; PS, performance status; RMST, restricted mean survival time; SABR, stereotactic ablative body radiotherapy; SBRT, stereotactic body radiotherapy; ST, systemic therapy; TKI, tyrosine kinase inhibitor; TTF, time-to-treatment failure; UCSD, urothelial carcinoma with squamous differentiation; UTUC, upper tract urothelial carcinoma.

## 4 Predictive biomarkers in urological cancer immunotherapy

A biomarker-driven approaches are transforming patient selection across all three tumor types.

### 4.1 Bladder cancer

#### 4.1.1 Circulating tumor DNA

The IMvigor011 trial established ctDNA as the most clinically advanced biomarker in MIBC. In this trial, patients with detectable ctDNA after RC had a poor prognosis (median DFS 4.8 months with placebo) but derived significant benefit from adjuvant atezolizumab (median DFS 9.9 months; HR 0.64). Importantly, patients with persistent ctDNA-negative status had excellent outcomes without adjuvant therapy (DFS 95% at 1 year, 88% at 2 years). This approach enables precision adjuvant therapy, identifying patients with molecular residual disease (MRD) who need treatment while sparing those already cured. ctDNA monitoring during treatment also showed that clearance of ctDNA was associated with response. The trial used two highly similar assays (Signatera from Natera in most countries, and an assay from BGI in China), demonstrating feasibility across different platforms. Limitations: ctDNA assays are not standardized across platforms; false-negative rates are ~15-20% (24).

#### 4.1.2 PD-L1 expression

The role of PD-L1 as a predictive biomarker in bladder cancer remains controversial. In the JAVELIN Bladder 100 trial, PD-L1 positivity (≥25% on tumor cells or ≥25% on immune cells if immune cells >1% of tumor area, or 100% on immune cells if immune cells ≤1%) was a stratification factor, and outcomes appeared similar regardless of PD-L1 status (31, 32). In the POTOMAC trial, PD-L1 status was not a selection criterion, and the benefit of durvalumab was observed across PD-L1 subgroups. However, in the ALBAN trial, PD-L1 expression was not associated with outcome. The lack of standardization of PD-L1 assays (SP142, 22C3, 28-8, SP263) and scoring algorithms (tumor proportion score, combined positive score, immune cell score) remains a major limitation (17, 18).

#### 4.1.3 Molecular subtypes

Transcriptomic subtyping of bladder cancer (luminal, basal, luminal infiltrated, etc.) is associated with differential ICI responses, but has not yet been prospectively validated in RCTs. The TCGA classification and consensus subtypes may eventually guide therapy selection (124, 125).

### 4.2 Kidney cancer

#### 4.2.1 Transcriptomic signatures

The IMmotion150 trial identified three gene expression signatures, angiogenesis, T-effector/IFN-γ response, and myeloid inflammation, that were differentially associated with PFS across treatments. The T-cell-inflamed GEP (TcellinfGEP) was positively associated with outcomes in the pembrolizumab plus axitinib arm of KEYNOTE-426 (ORR p<0.0001, PFS p<0.0001, OS p=0.002) but not in the sunitinib arm, supporting its role as a predictive biomarker for pembrolizumab response. The angiogenesis signature was positively associated with outcomes in the sunitinib arm (ORR p=0.002, PFS p<0.001, OS p<0.001). These signatures have been incorporated into molecular subtyping algorithms (angiogenic, immune-proliferative, etc.) that may guide treatment selection in the future (48, 54).

#### 4.2.2 Kidney injury molecule-1

In the IMmotion010 trial, plasma KIM-1 emerged as the most promising serum biomarker for minimal residual disease. KIM-1 is overexpressed in clear cell and papillary RCC, and its extracellular domain is shed into the circulation. High baseline KIM-1 (≥86 pg/mL) identified patients with poor prognosis (DFS HR 1.68) who derived benefit from adjuvant atezolizumab (HR 0.72), while low KIM-1 patients did not benefit (HR 1.20). An increase in KIM-1 during follow-up was associated with recurrence, particularly in placebo-treated KIM-1-high patients (median DFS 4.8 months) compared with atezolizumab-treated patients (14.8 months). KIM-1 kinetics thus represents a dynamic biomarker for treatment monitoring. However, the cutoff was derived *post-hoc* and requires prospective validation; the assay is not widely available. These findings require prospective validation but suggest that KIM-1 could guide adjuvant therapy decisions (39).

#### 4.2.3 PD-L1 expression

PD-L1 is not a reliable predictive biomarker in RCC. In KEYNOTE-426, PD-L1 CPS was not associated with outcomes in the pembrolizumab plus axitinib arm. In CheckMate 214, PD-L1 expression on tumor cells (≥1%) was associated with higher ORR with nivolumab plus ipilimumab (58% vs. 37%) but did not affect sunitinib outcomes, yet the OS benefit was observed regardless of PD-L1 status. The variability in PD-L1 assays and cutoffs, as well as the dynamic nature of PD-L1 expression, limit its clinical utility (42–44).

#### 4.2.4 DNA alterations

Mutations in PBRM1, SETD2, BAP1, and VHL have been studied as potential biomarkers. In KEYNOTE-426, PBRM1 mutation was associated with improved ORR in the pembrolizumab plus axitinib arm (71.4% vs. 52.3%; p=0.002). In the sunitinib arm, VHL and PBRM1 mutations were associated with longer OS (p=0.040 and p=0.010, respectively), while BAP1 mutation was associated with shorter OS (p=0.019). However, the totality of evidence suggests that single-gene alterations are not sufficiently predictive, and composite biomarkers (e.g., PBRM1-BAP1 mutation status) may be more informative (48).

#### 4.3.5 LAG-3 expression

In the FRACTION-RCC trial, higher baseline LAG-3 expression was observed among responders to nivolumab plus relatlimab. LAG-3 is an immune checkpoint that is co-expressed with PD-1 on exhausted T cells, and dual targeting may be effective in LAG-3-expressing tumors (51).

### 4.3 Prostate cancer

#### 4.3.1 PD-L1 expression

PD-L1 expression is low in most prostate cancers, limiting its utility as a predictive biomarker. In CheckMate 650, patients with PD-L1 ≥1% had numerically higher ORR (36.4% vs. 12.1%) and longer rPFS (5.6 vs. 3.9 months) with nivolumab plus ipilimumab (69). In the durvalumab plus tremelimumab trial, responding tumors were PD-L1-positive (70). However, the small numbers of responders preclude definitive conclusions.

#### 4.3.2 DNA damage repair defects

DDR mutations (e.g., BRCA1/2, ATM, CDK12) are enriched in prostate cancer and are associated with higher TMB and neoantigen load. In CheckMate 650, patients with HRD-positive or DDR-positive tumors had higher ORR (50.0% vs. 22.6% for HRD; 36.4% vs. 23.1% for DDR) and longer rPFS and OS (69). In the durvalumab plus tremelimumab trial, responses were enriched in tumors with DDR defects (70). CDK12 inactivation has been specifically associated with sensitivity to ICI in prostate cancer. These findings support the use of DDR testing for patient selection.

#### 4.3.3 Tumor mutational burden

In CheckMate 650, patients with TMB above the median had higher ORR (50.0% vs. 5.3%), PSA response rate (30.0% vs. 5.9%), and longer rPFS (7.4 vs. 2.4 months) and OS (19.0 vs. 10.1 months). However, TMB is generally low in prostate cancer, limiting the fraction of patients with high TMB (69).

#### 4.3.4 Microsatellite instability (MSI-high)/mismatch repair deficiency

MSI-high/dMMR occurs in only 2-5% of prostate cancers but is associated with high response rates to ICI (ORR ~40-60%). MSI-high/dMMR status should be tested in all patients with advanced prostate cancer, as it is an FDA-approved biomarker for pembrolizumab regardless of tumor type.

#### 4.3.5 Neutrophil-to-lymphocyte ratio and circulating immune subsets

In the phase III PPV trial, patients with lower baseline neutrophil proportion (<64%) or higher lymphocyte proportion (≥26%) derived significant OS benefit from PPV (HR 0.55 and 0.70, respectively). These simple blood biomarkers may reflect the balance between immunosuppressive MDSCs and effector lymphocytes (126).

## 5 Discussion

### 5.1 Implications for clinical practice by tumor type

**Figure 3:**
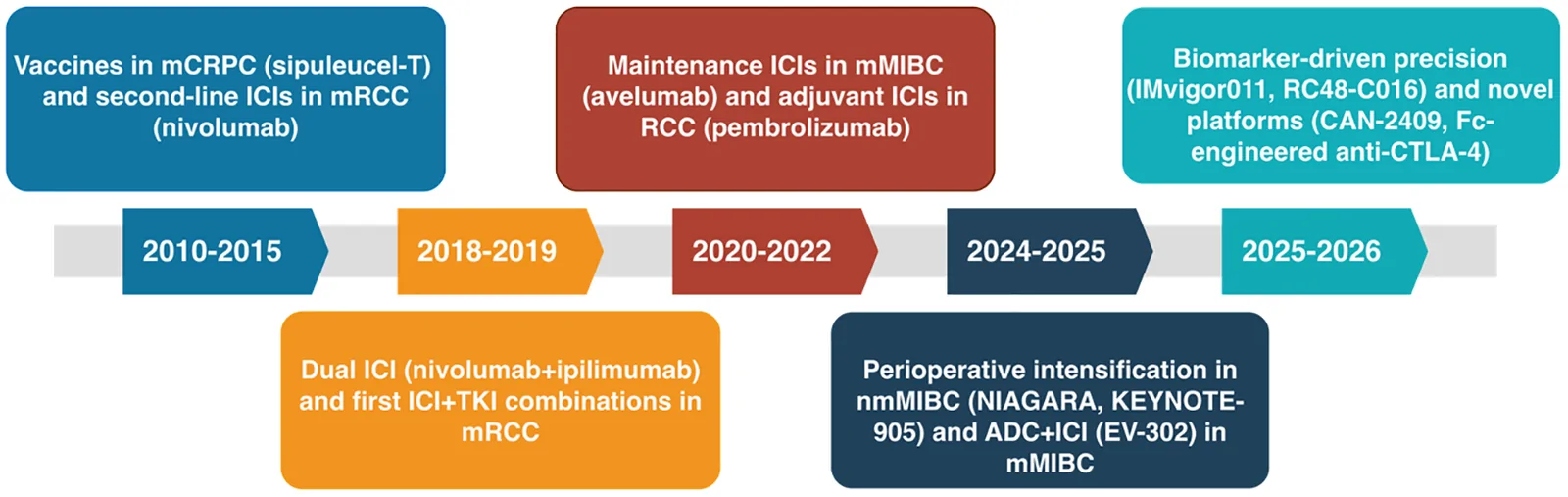
Chronological evolution of practice-changing phase III immunotherapy trials across bladder, kidney, and prostate cancers (2010–2026). This timeline illustrates the successive paradigm shifts in immunotherapy for urological malignancies, organized by tumor type. Bladder cancer progressed from maintenance therapy (avelumab, 2020–2022) to perioperative intensification with chemoimmunotherapy (NIAGARA, 2024) and ADC-ICI combinations (EV-302, 2025), culminating in biomarker-driven precision therapy (IMvigor011, RC48-C016) and chemotherapy-free perioperative regimens (KEYNOTE-905, 2026). Kidney cancer evolved from second-line monotherapy (nivolumab, 2015) through dual checkpoint blockade (nivolumab+ipilimumab, 2018), multiple ICI-TKI combinations (2019–2022), and adjuvant immunotherapy (pembrolizumab, 2022), to post-ICI strategies and alternative adjuvant regimens (2025–2026). Prostate cancer saw the first therapeutic vaccine (sipuleucel-T, 2010), subsequent vaccine failures (PROSTVAC, 2019), and recent emergence of novel platforms, including viral immunotherapy (CAN-2409, 2025), dual ICI in molecularly selected patients (CheckMate 650, 2025), and Fc-engineered checkpoint inhibitors (NeoRED-P, 2026). Abbreviations: ADC, antibody-drug conjugate; ICI, immune checkpoint inhibitor; nmMIBC, non-metastatic muscle-invasive bladder cancer; TKI, tyrosine kinase inhibitor; TRAE.

The past sixteen years have witnessed a remarkable evolution in immunotherapy for urological cancers, transitioning from first-generation vaccines to sophisticated biomarker-guided combination strategies. **Figure 3** illustrates this chronological progression of practice-changing phase III innovations across bladder, kidney, and prostate malignancies, highlighting the successive paradigm shifts that have transformed patient management.

#### 5.1.1 Bladder cancer

In NMIBC, the divergent results of POTOMAC (positive with durvalumab plus BCG) and ALBAN (negative with atezolizumab plus BCG) highlight that not all ICI-BCG combinations are effective. The 2-year BCG maintenance schedule in POTOMAC (versus 1 year in ALBAN) may be critical, as may the specific ICI agent. POTOMAC supports durvalumab plus BCG as a potential new treatment, but regulatory approval will depend on further data (17, 18).

In MIBC, perioperative durvalumab plus neoadjuvant chemotherapy (NIAGARA) has expanded curative-intent options for cisplatin-eligible patients. The 24-month EFS improvement of 8% and OS improvement of 7% are clinically meaningful. However, the optimal patient population remains to be defined, as the trial was not designed to isolate the contribution of neoadjuvant versus adjuvant phases. The FDA approval noted this limitation, and ongoing trials are investigating whether adjuvant therapy alone might suffice for patients achieving a pCR, or whether NAT alone could be sufficient for those with favourable biomarkers (21).

For the large proportion of patients who are cisplatin-ineligible or decline cisplatin-based chemotherapy, the KEYNOTE-905/EV-303 trial demonstrated that perioperative EV plus pembrolizumab dramatically improves EFS (HR 0.40), OS (HR 0.50), and pCR (57.1% vs. 8.6%) compared with surgery alone. This represents a transformative, curative-intent, bladder-sparing-compatible regimen for a previously underserved population (22).

In la/mUC, first-line EV plus pembrolizumab (EV-302/KEYNOTE-A39) has become standard of care option, nearly doubling median OS (33.8 vs. 15.9 months) and improving PFS (HR 0.48) regardless of cisplatin eligibility or PD-L1 expression (28). For patients with HER2-expressing tumours (IHC 1+, 2+, 3+), the combination of DV plus toripalimab (RC48-C016) offers a highly effective biomarker-driven option (PFS HR 0.36; OS HR 0.54) (29). Avelumab maintenance after platinum-based chemotherapy (JAVELIN Bladder 100) remains standard for patients without progression, with long-term data confirming sustained OS benefits, particularly in older patients and those with non-visceral metastases (31, 32).

ctDNA-guided adjuvant atezolizumab (IMvigor011) introduces precision adjuvant therapy, identifying patients with molecular residual disease who benefit from immunotherapy while sparing low-risk patients unnecessary treatment, a paradigm now being adopted across tumour types (24).

#### 5.1.2 Kidney cancer

Long-term follow-up of CheckMate 214 (nivolumab+ipilimumab), CheckMate 9ER (nivolumab+cabozantinib), KEYNOTE-426 (pembrolizumab+axitinib), CLEAR (lenvatinib+pembrolizumab), and JAVELIN Renal 101 (avelumab+axitinib) confirms durable survival benefits for ICI-based combinations, with 5-year OS rates of approximately 40–50% in intermediate/poor-risk patients. The choice of first-line therapy now depends on multiple factors: IMDC risk group (favourable-risk patients may not derive OS benefit from dual ICI, although PFS and ORR benefits exist), presence of sarcomatoid features (dual ICI particularly effective), tolerability profile, and patient preference (**Table 6**) (42–50). The FRACTION RCC trial suggests that LAG-3 inhibition (nivolumab+relatlimab) may offer a better safety profile than CTLA-4 blockade, though with numerically lower ORR (51). The negative NIVOSWITCH trial demonstrates that switch maintenance to ICI after TKI induction is not beneficial; patients responding to TKI should continue TKI (52).

**Table 6:** Comparative summary and clinical implications of first-line regimens in metastatic clear cell RCC.

Regimen	Key strength	Key limitation	Preferred patient population
Nivolumab + Ipilimumab (43, 44)	Superior sarcomatoid efficacy (HR 0.46); TFS advantage (10.1 vs 4.1 mo at 60 mo); chemotherapy-free	No OS benefit in favourable risk (HR 0.94); higher irAE profile	Intermediate/poor risk; sarcomatoid features
Nivolumab + Cabozantinib (45–47)	High CR rate (13.9%); balanced efficacy; 22% response durability at 60 mo	Favorable risk: no OS benefit (HR 1.08); cabozantinib toxicity (hypertension, PPE, diarrhoea)	All-risk (except favourable if OS preferred)
Pembrolizumab + Axitinib (48)	Longest follow-up (5-yr); biomarker insights (TcellinfGEP, PBRM1); 5-yr OS HR 0.84	Axitinib-specific toxicity; 63% grade ≥3 TRAEs	All-risk; biomarker-driven selection possible
Lenvatinib + Pembrolizumab (49)	Highest PFS benefit (HR 0.39) and ORR (71%); 16.1% CR; activity across all metastatic sites	Highest grade ≥3 TRAEs (72%); lenvatinib-related hypertension, diarrhoea, PPE	All-risk; patients tolerating aggressive TKI
Avelumab + Axitinib (50)	PD-L1+ efficacy confirmed (PFS HR 0.61)	OS data immature; PD-L1-driven primary analysis	PD-L1+ tumours; alternative PD-L1 inhibitor option

CR, complete response; HR, hazard ratio; irAE, immune-related adverse event; mo, months; ORR, objective response rate; OS, overall survival; PD-L1, programmed death-ligand 1; PPE, palmar-plantar erythrodysesthesia; RCC, renal cell carcinoma; TFS, treatment-free survival; TRAE, treatment-related adverse event.

In the adjuvant setting, pembrolizumab (KEYNOTE-564) is the standard for high-risk resected RCC, with demonstrated DFS benefit and emerging OS benefit (35–37). The RAMPART trial offers an alternative with durvalumab plus tremelimumab, albeit with higher toxicity (38). Biomarkers such as KIM-1 (IMmotion010) are moving toward clinical utility, potentially enabling biomarker-driven adjuvant therapy selection (39).

Sequencing after ICI progression is now informed by the FRUSICA-2 trial, establishing fruquintinib plus sintilimab as an effective second-line option (PFS HR 0.373) (41). Emerging data from the OPTIC RCC trial suggest the feasibility of transcriptomic phenotype-guided therapy (angiogenic/stromal vs. immune clusters), moving beyond clinical risk stratification toward molecularly guided first-line selection (55).

#### 5.1.3 Prostate cancer

Despite historically modest responses to ICI monotherapy, combination strategies are emerging. CheckMate 650 shows activity of dual ICI (nivolumab+ipilimumab) in selected patients, particularly those with DDR defects, high TMB, or MSI-high status. However, significant toxicity (grade 3–4 TRAEs in 42–53%, four treatment-related deaths) mandates careful patient selection (69). The CA184-043 trial showed long-term survival benefits with ipilimumab plus radiotherapy, with 5-year OS rates of 7.9% vs. 2.7%, although the early hazard was increased (82).

Cancer vaccines remain an option for asymptomatic or minimally symptomatic mCRPC. Sipuleucel-T (IMPACT) is supported by long-term data showing durable responses and antigen spread, with evidence supporting earlier use (lower baseline PSA, better prognostic features) (1, 71–74). Newer vaccine approaches, such as the telomerase-based GX301 vaccine and the PPV, have shown promise in phase II trials but require phase III confirmation. The negative phase III PPV trial and the DCVAC/PCa trial highlight the difficulty of translating vaccine efficacy.

Oligometastatic disease: The EXTEND trial establishes MDT plus ADT as a new option, improving median PFS from 17 to 36 months (HR 0.45) (65). This is the largest randomised trial of MDT in prostate cancer and the first to show improvements in radiographic PFS and castration-resistance-free survival, with immune correlates (TCR expansion/contraction) providing a mechanistic rationale.

Novel immunotherapies: The NeoRED-P trial provides the first-in-human evidence that Fc-engineered anti-CTLA-4 can deplete TI-Tregs in prostate cancer when combined with ADT, with associated DFS benefit. This addresses the failure of first-generation anti-CTLA-4 antibodies to deplete Tregs and suggests a path forward for CTLA-4 blockade in prostate cancer (64). The CAN-2409 trial (viral immunotherapy) demonstrated improved DFS (HR 0.70) and increased pCRs when added to radiotherapy in localized intermediate-/high-risk disease (62). The bispecific T-cell engager pasritamig (KLK2xCD3) showed a PSA50 response rate of 42.4% in heavily pre-treated mCRPC, opening a new class of immunotherapy for prostate cancer (68).

### 5.2 Common themes across malignancies

#### 5.2.1 Toxicity profiles across immunotherapeutic classes

Management in comorbid populations: Patients with pre-existing autoimmune disease had higher rates of grade ≥3 irAEs (35% vs. 18%) but derived similar OS benefit. Patients with chronic kidney disease receiving EV+pembrolizumab had higher rates of acute kidney injury (15% vs. 5%).

#### 5.2.2 Biomarker-driven therapy is becoming reality

ctDNA guidance for adjuvant atezolizumab in MIBC (IMvigor011) now allows treatment of molecular residual disease while sparing patients without detectable ctDNA. In RCC, plasma KIM-1 (IMmotion010) shows promise for identifying patients who benefit from adjuvant atezolizumab, and transcriptomic signatures (angiogenic, T-effector, myeloid inflammation) from IMmotion150 and OPTIC RCC are moving toward clinical application (39, 54, 55). PD-L1 expression, while not a reliable predictor in RCC (KEYNOTE-426, CheckMate 214), has been explored in bladder and prostate cancer (42–44, 48). DDR defects, TMB, and MSI-high status identify a subset of prostate cancer patients who may respond to dual ICI (CheckMate 650).

#### 5.2.3 Immunotherapy is moving to earlier disease stages

Perioperative approaches are now standard in MIBC for both cisplatin-eligible (durvalumab, NIAGARA) and cisplatin-ineligible (EV plus pembrolizumab, KEYNOTE-905/EV-303) patients (21, 22). In RCC, adjuvant pembrolizumab (KEYNOTE-564) and the durvalumab/tremelimumab combination (RAMPART) reduce recurrence risk after nephrectomy (35–38). Neoadjuvant Fc-enhanced anti-CTLA-4 plus ADT (NeoRED-P) has shown proof-of-principle in high-risk localized prostate cancer (64). The rationale is that the immune system is more intact in earlier disease, and eliminating minimal residual disease may be more effective than treating bulky metastases.

#### 5.2.4 Combination strategies are the norm – but with exceptions

Single-agent immunotherapy is rarely sufficient. In MIBC, ICI is combined with chemotherapy (NIAGARA), with BCG (POTOMAC), or with ADCs (EV-302, RC48-C016, KEYNOTE-905) (17, 21, 22, 28). In RCC, ICI is combined with another ICI (CheckMate 214, FRACTION RCC) or with TKIs (CheckMate 9ER, KEYNOTE-426, CLEAR, JAVELIN Renal 101) (42, 47, 50, 51). In prostate cancer, vaccines are combined with ADT (sipuleucel-T), ICI with radiotherapy (CA184-043), and ADT with Fc-enhanced anti-CTLA-4 (NeoRED-P) (1, 65, 82). However, the negative NIVOSWITCH trial warns against switching from TKI to ICI maintenance in responding patients, and the DISCUS trial suggests that abbreviated chemotherapy (3 cycles) followed by avelumab maintenance may be sufficient in aUC, preserving quality of life without compromising survival (33, 52).

### 5.3 Treatment optimization and de-escalation are gaining traction

ctDNA-guided adjuvant therapy (IMvigor011) and abbreviated chemotherapy (DISCUS, 3 vs. 6 cycles) exemplify efforts to reduce treatment burden while maintaining efficacy (24, 33, 56). Extended interval ICI dosing was shown to be safe in CheckMate 800 (56). The intermittent short-course enzalutamide approach in nmHSPC offers prolonged PSA control with minimal toxicity, potentially deferring the need for continuous ADT (67). In UTUC, the DISTINCT-I trial supports nephron-sparing strategies with ADC/ICI combinations, challenging radical nephroureterectomy as the sole standard (34).

### 5.4 Safety profiles vary significantly across classes and combinations

ICI combinations containing CTLA-4 blockade have substantial toxicity (grade 3–4 TRAEs in 42–53% in CheckMate 650; 38% in RAMPART) (38, 59). ICI-TKI combinations show grade 3–4 TRAEs in 40–71% (71.2% for avelumab+axitinib in JAVELIN Renal 101) (51). In contrast, cancer vaccines (sipuleucel-T, PROSTVAC, PPV, GX301) are generally well tolerated, with injection-site reactions and flu-like symptoms being most common (1, 66, 78). Novel agents such as the bispecific T-cell engager pasritamig show manageable cytokine release syndrome (68). The choice of therapy must balance efficacy and toxicity, particularly in asymptomatic patients and those with multiple comorbidities (**Table 7**).

**Table 7:** Grade ≥3 treatment-related adverse events by regimen class.

Class	Regimen (trial)	Grade ≥3 TRAEs	Common toxicities	Treatment-related deaths
Dual ICI (43)	Nivolumab+ipilimumab	42-53%	Colitis (11%), hepatitis (9%)	1.4%
ADC +ICI (28)	EV+pembrolizumab	55.5%	Rash (18%), peripheral neuropathy (12%)	0.1%
ADC+ICI (29)	Disitamab vedotin+toripalimab	55%	Neutropenia (18%)	1.2%
ICI+TKI (48)	Pembrolizumab+axitinib	75%	Hypertension (22%), diarrhea (11%)	0.7%
Vaccine (1)	Sipuleucel-T	3%	Chills, fever (grade 1-2)	0%

### 5.5 Novel mechanisms are expanding the immunotherapy toolkit beyond classical checkpoints

The phase II GDFather-NEO trial provides proof-of-concept for targeting the GDF-15 resistance pathway in MIBC, tripling the pCR rate when added to neoadjuvant nivolumab (25). The bispecific T-cell engager pasritamig opens a new class for prostate cancer (68). The viral immunotherapy CAN-2409 improves DFS in localized prostate cancer when added to radiotherapy (62). LAG-3 inhibition (nivolumab+relatlimab) shows activity in RCC with a more favourable safety profile than CTLA-4 blockade (FRACTION RCC) (51). These emerging mechanisms are likely to diversify the immuno-oncology landscape in the coming years.

### 5.6 Immunoradiotherapy: An emerging paradigm with distinct challenges

The integration of radiotherapy with immunotherapy represents one of the most promising yet challenging frontiers in urological oncology. The biological rationale is compelling: radiation-induced immunogenic cell death provides an “*in situ* vaccination” effect, while checkpoint inhibition prevents T-cell exhaustion, potentially yielding synergistic antitumour activity. However, the clinical data reveal a more nuanced picture.

In bladder cancer, bladder-preserving immunoradiotherapy has demonstrated remarkable efficacy, with CR rates of 64-88% across multiple phase II trials (1, 3, 7) (85, 87, 92). The consistently high CR rates suggest that this approach may offer a genuine alternative to radical cystectomy for selected patients, particularly those who are cisplatin-ineligible or elderly. However, the toxicity profile requires careful attention: the PCR-MIB trial reported 32% unacceptable toxicity (87), and the PLUMMB trial was terminated for dose-limiting urinary toxicity with hypofractionated regimens (89). These findings underscore the critical importance of fractionation selection, with conventional fractionation (55–65 Gy) appearing safer than hypofractionated schedules (36 Gy in 6 fractions) in the bladder.

The INDIBLADE trial’s identification of ctDNA clearance as a predictive biomarker represents a significant advance (86). Patients clearing ctDNA after induction immunotherapy had substantially better outcomes, suggesting that this biomarker could guide treatment intensification or de-escalation. This parallels the ctDNA-guided approach validated in the IMvigor011 trial (24), reinforcing the emerging paradigm of molecular response-directed therapy.

In prostate cancer, the CA184–043 trial’s long-term data demonstrated that a subset of patients derive durable benefit from ipilimumab plus radiotherapy, despite early toxicity (97). The crossing survival curves, with early excess mortality in the ipilimumab arm followed by sustained separation, illustrate the importance of long-term follow-up in immuno-oncology trials. The Bryant trial’s validation of the Decipher immunosuppression score as a predictive biomarker (98) offers a pathway to patient selection, potentially identifying those most likely to benefit from immunoradiotherapy intensification.

In oligometastatic RCC, the RAPPORT trial’s 63% ORR and 92% local control (101) compare favourably with the NIVES study’s 17% ORR in more disseminated disease (102), suggesting that the extent of metastatic burden and the completeness of ablation are critical determinants of efficacy. This aligns with the EXTEND trial’s findings in oligometastatic prostate cancer, where metastasis-directed therapy improved outcomes (65).

Key unanswered questions persist: What is the optimal radiation dose and fractionation for each tumour type and anatomical site? Should radiotherapy be delivered concurrently or sequentially with immunotherapy? Which patients are most likely to benefit, and which biomarkers, ctDNA, transcriptomic signatures, or immune cell subsets, can guide selection? The ongoing phase I triplet trials with atezolizumab, tiragolumab, and SBRT may provide insights into multi-agent immunoradiotherapy combinations (104). Prospective validation of biomarkers and randomized comparisons of different sequencing strategies are urgently needed before immunoradiotherapy can be widely adopted into routine practice.

### 5.7 Real-world evidence: Bridging the efficacy-effectiveness gap

The integration of real-world evidence has become essential to complement the controlled findings of randomized trials. Across urological malignancies, several critical themes emerge from RWD analyses that directly inform clinical practice.

The performance status conundrum: Real-world populations consistently present with worse performance status than trial cohorts, and ECOG PS ≥2 emerges as one of the most powerful negative prognostic factors across all tumour types. The Dutch nationwide comparison of pembrolizumab in la/mUC demonstrated that despite comparable ORR, median OS was nearly halved in real-world populations (5.5 vs. 10.1 months) due to higher proportions of patients with poor PS, anaemia, and prior carboplatin exposure (2). This highlights the critical importance of patient selection and the limitations of extrapolating trial outcomes to frail populations.

Histologic heterogeneity matters: The ARON-2EV study established urothelial carcinoma with squamous differentiation as an independent adverse prognostic factor across all modern systemic therapies, with consistently shorter OS regardless of treatment modality (105). This finding underscores the need for histologic subtyping in both trial design and clinical decision-making, as outcomes in variant histologies may differ substantially from pure urothelial carcinoma.

Treatment-free intervals and sequencing: The Dutch real-world comparison of maintenance avelumab versus pembrolizumab at progression suggests that a treatment-free interval after chemotherapy, followed by ICI at progression, may be as effective as immediate maintenance therapy while minimizing toxicity and treatment burden (106). This challenges the assumption that earlier is always better and supports the concept of strategic treatment breaks. Similarly, the ICI rechallenge data from the TriNetX analysis suggest that patients with longer treatment-free intervals (>12 months) derive substantially greater benefit from rechallenge, highlighting the potential for immune recovery and restoration of checkpoint sensitivity over time (112).

Immuno-radiotherapy synergy in practice: The ARON-2 study’s finding of improved 1-year OS with SBRT plus pembrolizumab (61% vs. 47%) (110) and the RAPPORT trial’s 63% ORR in oligometastatic RCC (116) provide real-world validation of the preclinical synergy between radiotherapy and immunotherapy. These data support the ongoing integration of ablative radiotherapy into immunotherapy protocols, particularly in oligometastatic settings where complete ablation of all visible disease may be achievable.

Access and adoption gaps: The Japanese perioperative study revealed stark differences in immunotherapy adoption between MIBC (75.4% of adjuvant treatments) and UTUC (0.9%), despite similar biological rationale (119). This disparity likely reflects differences in practice patterns, reimbursement, and provider familiarity, highlighting the need for greater dissemination of evidence and guideline implementation across tumour subtypes.

The value of combination with hormonal therapy: In prostate cancer, the Medicare analysis demonstrating superior OS with Sipuleucel-T plus ARTA compared to ARTA alone supports the biological rationale that androgen deprivation enhances immune responses by modulating the tumour microenvironment. This combination strategy may offer a template for integrating immunotherapy earlier in the prostate cancer treatment paradigm.

Ultimately, real-world evidence serves as an essential bridge between the controlled environment of RCTs and the complexity of routine clinical practice. Prospective registries such as PROCEED and AVENANCE (108), alongside retrospective cohort analyses, provide critical insights into treatment patterns, access disparities, and outcomes in populations systematically excluded from trials. As immunotherapy continues to evolve, the integration of RWD with RCT data will be essential to define optimal treatment sequences, identify patients most likely to derive long-term benefit, and ensure equitable access across diverse healthcare settings.

### 5.8 Limitations of the evidence

#### 5.8.1 Immature follow-up

Several practice-changing trials included in this review report outcomes with relatively short median follow-up (e.g., NIAGARA and KEYNOTE-905/EV-303 at 25.6 months; EV-302 at 29.1 months). The long-term durability of EFS and OS benefits beyond 2–3 years remains unknown, and OS data may attenuate with longer follow-up (21, 22).

#### 5.8.2 Selection bias

RCTs enroll younger, fitter patients. Patients with eGFR <30 mL/min or history of autoimmune disease were excluded.

#### 5.8.3 Cross-trial comparability

Direct head-to-head comparisons between different ICI-based regimens are lacking. Cross-trial comparisons are confounded by differences in patient populations and control arms.

#### 5.8.4 Crossover effects

In CheckMate 214, 42% of sunitinib-arm patients received subsequent ICI therapy, potentially diluting the OS benefit (43).

#### 5.8.5 Evolving standards of care

Some trials used control arms that are no longer standard (e.g., platinum-based chemotherapy for la/mUC is no longer first-line since EV-302).

#### 5.8.6 Caution regarding recent data

Several trials included in this review (EV-302 2.5-year update, RC48-C016, KEYNOTE-905/EV-303, IMvigor011, FRUSICA-2) reported data from 2025–2026 with limited follow-up. OS benefits may attenuate with longer follow-up, and unexpected late toxicities cannot be excluded (22, 25).

#### 5.8.7 Certainty of evidence

Formal GRADE assessment was not performed for all comparisons due to the narrative scope. However, key limitations are acknowledged.

#### 5.8.8 Lack of PROSPERO registration

Prospective registration of systematic review protocols is recommended. The absence of registration is acknowledged as a limitation.

### 5.9 Future directions

Key unanswered questions and future directions include:

#### 5.9.1 Validation of biomarkers

ctDNA, KIM-1, transcriptomic signatures, and other promising biomarkers require prospective validation in large cohorts. The development of standardized, commercially available assays is essential for clinical implementation.

#### 5.9.2 Novel combinations to overcome resistance

Rational combinations based on resistance mechanisms are needed. For ICI-resistant tumors, combining with oncolytic viruses, CAR-T cells, or novel immunomodulators (e.g., STING agonists, TLR agonists) may restore sensitivity.

#### 5.9.3 Expansion of cellular therapies

CAR-T cells have revolutionized hematologic malignancies but have had limited success in solid tumors due to the immunosuppressive TME. Novel approaches (armored CAR-T cells, CAR-NK cells, allogeneic CAR-T cells) are in early development.

#### 5.9.4 Health-related quality of life and patient-reported outcomes

While some trials (JAVELIN Bladder 100, KEYNOTE-564) have reported PRO data, many have not. PROs should be standard endpoints in future trials.

#### 5.9.5 Cost-effectiveness analyses

With the high cost of immunotherapies, cost-effectiveness analyses are essential to inform healthcare policy and ensure equitable access.

#### 5.9.6 Identification of optimal sequencing

Prospective trials comparing different sequencing strategies (e.g., ICI first vs. TKI first vs. ADC first) are needed. Adaptive platform trials may efficiently address these questions.

#### 5.9.7 Development of effective immunotherapies for “cold” tumors

Prostate cancer remains the most challenging urological malignancy for immunotherapy. Novel approaches, including therapeutic vaccines, oncolytic viruses, and combinations with ADT or radiotherapy, are being explored.

## 6 Conclusions

Immunotherapy has revolutionized the management of bladder, kidney, and prostate cancers over the past decade and a half. From the first approval of sipuleucel-T in 2010 to the recent emergence of ADCs, bispecific T-cell engagers, and novel ICI combinations, the therapeutic landscape has continuously evolved.

In bladder cancer, current paradigms include: (1) For cisplatin-eligible MIBC, perioperative durvalumab plus neoadjuvant chemotherapy improves EFS and OS; (2) For cisplatin-ineligible or -declining MIBC, perioperative EV plus pembrolizumab dramatically improves EFS and pCR, offering a curative-intent option; (3) In la/mUC, first-line EV plus pembrolizumab has become the global standard, nearly doubling median OS (33.8 vs. 15.9 months). The biomarker-driven combination of disitamab vedotin plus toripalimab provides a highly effective first-line option for HER2-expressing tumours; (4) ctDNA-guided adjuvant atezolizumab introduces precision therapy for molecular residual disease after cystectomy; and (5) The negative ALBAN trial serves as a reminder that not all ICI-BCG combinations are effective in non-muscle-invasive disease.

In kidney cancer, ICI-based combinations have become the standard first-line therapy for advanced clear cell renal cell carcinoma, with long-term (≥5 years) survival benefits confirmed for nivolumab+ipilimumab, nivolumab+cabozantinib, pembrolizumab+axitinib, lenvatinib+pembrolizumab, and avelumab+axitinib. Adjuvant pembrolizumab reduces recurrence risk after nephrectomy for high-risk disease. Emerging biomarkers, including KIM-1, transcriptomic signatures (angiogenic, T-effector), and PBRM1 mutations, may guide future treatment selection. In the post-immunotherapy setting, fruquintinib + sintilimab offers a highly effective second-line option.

In prostate cancer, sipuleucel-T remains an option for asymptomatic or minimally symptomatic mCRPC, with evidence supporting earlier use. Dual ICI (nivolumab+ipilimumab) shows activity in molecularly selected patients (DNA damage repair defects, high TMB, MSI-H). The addition of metastasis-directed therapy to ADT improves progression-free survival in oligometastatic disease. Neoadjuvant Fc-enhanced anti-CTLA-4 demonstrates Treg depletion and improved DFS in high-risk localized prostate cancer, overcoming a limitation of first-generation anti-CTLA-4 antibodies. Novel immunotherapies such as the bispecific T-cell engager pasritamig and the viral immunotherapy CAN-2409 have shown promising early results. However, several phase III vaccine trials (PROSTVAC, DCVAC/PCa, and the personalized peptide vaccine) have been negative, highlighting the challenges of vaccine development in prostate cancer.

Successful integration of these complex and often expensive therapies into routine clinical practice requires careful patient selection, rigorous biomarker development, and healthcare system adaptation. Continued commitment to high-quality clinical research, including biomarker-driven trials, patient-reported outcome assessments, and cost-effectiveness analyses, will be essential to fully realize the promise of immunotherapy for all patients with urological cancers.

## Data Availability

The original contributions presented in the study are included in the article/**Supplementary Material**, further inquiries can be directed to the corresponding author/s.

## Glossary

ADC: Antibody-Drug Conjugate
ADCC: Antibody-Dependent Cellular
ADT: Androgen Deprivation Therapy
AE: Adverse Event
APC: Antigen-Presenting CellL
ARPI: Androgen Receptor Pathway Inhibitor
BEFS: Bladder Preservation Event-Free Survival
BIDFS: Bladder-Intact Disease-Free Survival
BSC: Best Supportive Care
CAR: Chimeric Antigen Receptor
CI: Confidence Interval
CIK: Cytokine-Induced Killer
CIS: Carcinoma In Situ
CN: Cytoreductive Nephrectomy
CPS: Combined Positive Score
CR: Complete Response
CRP: C Reactive Protein
CRPC: Castration-Resistant Prostate Cancer
CRS: Cytokine Release Syndrome
ctDNA: Circulating Tumor DNA
CTLA 4: Cytotoxic T Lymphocyte Associated Protein 4
DCR: Disease Control Rate
DDR: DNA Damage Repair
DFS: Disease Free Survival
DLT: Dose Limiting Toxicity
DMFS: Distant Metastasis Free Survival
dMMR: Mismatch Repair Deficiency
EBRT: External Beam Radiotherapy
EFS: Event Free Survival
ELISpot: Enzyme-Linked Immunospot
EV: Enfortumab Vedotin
FDA: Food and Drug Administration
FFBR: Freedom from Biochemical Recurrence
FFLP: Freedom from Local Progression
GC: Gemcitabine Cisplatin
GEP: Gene Expression Profile
GM CSF: Granulocyte Macrophage Colony Stimulating Factor
GRANT: Grade, Age, Nodes, Tumor Score
GU: Genitourinary
HDRBT: High Dose Rate Brachytherapy
HER2: Human Epidermal Growth Factor Receptor 2
HIF: Hypoxia Inducible Factor
HLA: Human Leukocyte Antigen
HR: Hazard Ratio
HRD: Homologous Recombination Deficiency
HSV tk: Herpes Simplex Virus Thymidine Kinase
hTERT: Human Telomerase Reverse Transcriptase
ICI: Immune Checkpoint Inhibitor
IFN γ: Interferon Gamma
IgG: Immunoglobulin G
IHC: Immunohistochemistry
IL 2: Interleukin 2
IL 7: Interleukin 7
IMDC: International Metastatic RCC Database Consortium
IO: Immuno Oncology
irAE: Immune Related Adverse Event
ITT: Intention To Treat
KIM 1: Kidney Injury Molecule 1
LAG 3: Lymphocyte Activation Gene 3
la/mUC: Locally Advanced/Metastatic Urothelial Carcinoma
MDSC: Myeloid Derived Suppressor Cell
MDT: Metastasis Directed Therapy
MeSH: Medical Subject Headings
MIBC: Muscle Invasive Bladder Cancer
MMAE: Monomethyl Auristatin E
Mo MDSC: Monocytic Myeloid Derived Suppressor Cell
MPR: Major Pathologic Response
MRD: Molecular Residual Disease
MSI high: Microsatellite Instability –
NAT: Neoadjuvant Therapy
NED: No Evidence of Disease
NK: Natural Killer
NMIBC: Non Muscle Invasive Bladder Cancer
NR: Not Reported
NSCLC: Non Small Cell Lung Cancer
ORR: Objective Response Rate
OS: Overall Survival
PAP: Prostatic Acid Phosphatase
PBRM1: Polybromo 1
PBMC: Peripheral Blood Mononuclear Cell
pCR: Pathological Complete Response
PD 1: Programmed Cell Death Protein 1
PD L1: Programmed Death Ligand 1
PFS: Progression Free Survival
PG: Paclitaxel Gemcitabine
PPE: Palmar Plantar Erythrodysesthesia
PPV: Personalized Peptide Vaccination
PRO: Patient Reported Outcome
PrCa: Prostate Cancer
PS: Performance Status
PSA: Prostate Specific Antigen
PSADT: Prostate Specific Antigen Doubling Time
Q TWiST: Quality Adjusted Time Without Symptoms or Toxicity
QoL: Quality of Life
RC: Radical Cystectomy
RCC: Renal Cell Carcinoma
RCT: Randomized Controlled Trial
RECIST: Response Evaluation Criteria in Solid Tumors
RFS: Recurrence Free Survival
rhIL 7: Recombinant Human Interleukin 7
RIS: Radiotherapy Immunosuppression Score
RMST: Restricted Mean Survival Time
rPFS: Radiographic Progression Free Survival
RT: Radiotherapy
RWD: Real World Data
RWE: Real World Evidence
SABR: Stereotactic Ablative Body Radiotherapy
SBRT: Stereotactic Body Radiotherapy
sRCC: Sarcomatoid Renal Cell Carcinoma
ST: Systemic Therapy
SWiM: Synthesis Without Meta analysis
TAM: Tumor Associated Macrophage
TCR: T Cell Receptor
TFS: Treatment Free Survival
TI Treg: Tumor Infiltrating Regulatory T Cell
TKI: Tyrosine Kinase Inhibitor
TLR: Toll Like Receptor
TMB: Tumor Mutational Burden
TME: Tumor Microenvironment
TRAE: Treatment Related Adverse Event
Treg: Regulatory T Cell
TTF: Time to Treatment Failure
TURBT: Transurethral Resection of Bladder Tumor
UC: Urothelial Carcinoma
UCSD: Urothelial Carcinoma with Squamous Differentiation
UTUC: Upper Tract Urothelial Carcinoma
VEGF: Vascular Endothelial Growth Factor
VEGFR: Vascular Endothelial Growth Factor Receptor
VHL: Von Hippel Lindau
