# Supplementary material for: Immunotherapy in urological cancers: new paradigms and a systematic review of clinical trials and real-world evidence
Source: Front Oncol. 2026 Aug 12;16:1871832. doi: 10.3389/fonc.2026.1871832 (PMC13506281; doi:10.3389/fonc.2026.1871832)
Supplement: Supplementary file 1 [file Table1.docx]

**PRISMA 2020 Checklist – Systematic Review**

| **Section/Topic** | **Item #** | **Checklist Item** | **Location in Manuscript** | **Reported?** |  |
| --- | --- | --- | --- | --- | --- |
| TITLE |  |  |  |  |  |
| Title | 1 | Identify the report as a systematic review. | Title page: "Immunotherapy in urological cancers: New paradigms and a systematic review of randomized controlled trials" | ✓ |  |
| ABSTRACT |  |  |  |  |  |
| Abstract | 2 | Structured summary (background, methods, results, discussion/conclusions). | Abstract (lines 31–58) | ✓ |  |
| INTRODUCTION |  |  |  |  |  |
| Rationale | 3 | Describe the rationale for the review. | Introduction, paragraphs 1–4 | ✓ |  |
| Objectives | 4 | Provide an explicit statement of the objectives. | End of Introduction (Section 1.4) | ✓ |  |
| METHODS |  |  |  |  |  |
| Eligibility criteria | 5 | Specify inclusion and exclusion criteria. | Section 2.2 | ✓ |  |
| Information sources | 6 | Specify databases, dates, and other sources. | Section 2.3 | ✓ |  |
| Search strategy | 7 | Present full search strategy (at least one database). | Section 2.3 + Supplementary File S2 | ✓ (with supplement) |  |
| Selection process | 8 | Specify methods used to decide whether a study met the inclusion criteria. | Section 2.4 | ✓ |  |
| Data collection process | 9 | Specify methods used to collect data from reports. | Section 2.4 | ✓ |  |
| Data items | 10 | List and define all outcomes for which data were sought. | Section 2.4 | ✓ |  |
| Study risk of bias assessment | 11 | Specify methods used to assess risk of bias. | Section 2.5 | ✓ |  |
| Effect measures | 12 | Specify for each outcome the effect measure(s) (e.g., risk ratio, mean difference) used. | Section 2.4 | ✓ |  |
| Synthesis methods | 13 | Describe processes used to synthesize results. | Section 2.5 | ✓ |  |
| Reporting bias assessment | 14 | Describe methods used to assess risk of bias due to missing results. | Section 2.5 | ✓ |  |
| Certainty assessment | 15 | Describe methods used to assess certainty (or confidence) in the body of evidence. | Section 2.5 (GRADE assessment) | ✓ |  |
| RESULTS |  |  |  |  |  |
| Study selection | 16 | Describe results of the search and selection process. | Section 3.1 + PRISMA flow diagram | ✓ |  |
| Study characteristics | 17 | Cite each included study and present its characteristics. | Tables 1–5 | ✓ |  |
| Risk of bias in studies | 18 | Present assessments of risk of bias for each included study. | Section 2.5 (summary) + Supplementary File | ✓ |  |
| Results of individual studies | 19 | For all outcomes, present, for each study, summary statistics. | Section 3.2–3.6 (narrative) + Tables 1–5 | ✓ |  |
| Results of syntheses | 20 | Present results of each synthesis. | Section 3.2–3.6 | ✓ |  |
| Reporting biases | 21 | Present assessments of reporting bias. | Section 5.7 | ✓ |  |
| Certainty of evidence | 22 | Present assessments of certainty (or confidence) in the body of evidence. | Section 5.7.7 | ✓ |  |
| DISCUSSION |  |  |  |  |  |
| Discussion | 23 | Provide a general interpretation of the results. | Section 5.1–5.7 | ✓ |  |
| Limitations | 24 | Discuss limitations of the evidence. | Section 5.7 (including PROSPERO registration absence) | ✓ |  |
| Conclusions | 25 | Provide a general interpretation of the results. | Section 6 | ✓ |  |
| OTHER |  |  |  |  |  |
| Registration | 26 | Provide registration information. | Section 2.1 | ✗ *(Not registered; acknowledged as limitation in Section 5.7.8)* |  |
| Protocol | 27 | Indicate where the protocol can be accessed. | Section 2.1 (not available) | ✗ *(No protocol registered)* |  |
